# Ultrasound-induced single-electron reduction of azide groups in aromatic prodrugs

**DOI:** 10.1093/nsr/nwaf140

**Published:** 2025-04-10

**Authors:** Jiali Sun, Haochen Yao, Chenguang Yang, Fuxin Xue, Xitong Ren, Linjie Cui, Zhilin Liu, Zhaohui Tang, Xuesi Chen

**Affiliations:** State Key Laboratory of Polymer Science and Technology, Changchun Institute of Applied Chemistry, Chinese Academy of Sciences, Changchun 130022, China; School of Applied Chemistry and Engineering, University of Science and Technology of China, Hefei 230026, China; Key Laboratory of Zoonosis Research, Ministry of Education, College of Basic Medical Science, Jilin University, Changchun 130021, China; Hepatobiliary and Pancreatic Surgery Department, General Surgery Center, First Hospital of Jilin University, Changchun 130021, China; State Key Laboratory of Polymer Science and Technology, Changchun Institute of Applied Chemistry, Chinese Academy of Sciences, Changchun 130022, China; Department of Radiation Oncology, China-Japan Union Hospital of Jilin University, Changchun 130033, China; State Key Laboratory of Polymer Science and Technology, Changchun Institute of Applied Chemistry, Chinese Academy of Sciences, Changchun 130022, China; School of Applied Chemistry and Engineering, University of Science and Technology of China, Hefei 230026, China; State Key Laboratory of Polymer Science and Technology, Changchun Institute of Applied Chemistry, Chinese Academy of Sciences, Changchun 130022, China; School of Applied Chemistry and Engineering, University of Science and Technology of China, Hefei 230026, China; State Key Laboratory of Polymer Science and Technology, Changchun Institute of Applied Chemistry, Chinese Academy of Sciences, Changchun 130022, China; State Key Laboratory of Polymer Science and Technology, Changchun Institute of Applied Chemistry, Chinese Academy of Sciences, Changchun 130022, China; School of Applied Chemistry and Engineering, University of Science and Technology of China, Hefei 230026, China; State Key Laboratory of Polymer Science and Technology, Changchun Institute of Applied Chemistry, Chinese Academy of Sciences, Changchun 130022, China; School of Applied Chemistry and Engineering, University of Science and Technology of China, Hefei 230026, China

**Keywords:** ultrasound, azide, reduction, selective therapy

## Abstract

Ultrasound has emerged as a versatile clinical tool due to its high tissue penetration and ability to deliver energy precisely to targeted areas. Increasing attention is now focused on its ability to induce chemical transformations of compounds *in vivo* and the underlying mechanisms therein. Here, we report a new ultrasonic chemistry mechanism in which low-intensity ultrasound facilitates the single-electron reduction of aromatic azides, converting them to bioactive amines. This transformation is especially efficient with aromatic compounds bearing strong electron-withdrawing groups or minimal natural charge. Using azide-masked resiquimod (R848-N_3_) as a model, we demonstrate that ultrasound induces a reduction reaction mediated by β-nicotinamide adenine dinucleotide disodium salt hydrate, with riboflavin tetrabutyrate serving as an electron transfer catalyst, significantly enhancing reaction rates. In a colon cancer model, nanoparticles co-loaded with R848-N_3_ and riboflavin tetrabutyrate achieved a remarkable 99.0% tumor suppression rate and a 66.7% cure rate when paired with low-intensity ultrasound. This study reveals ultrasound as an effective switch for prodrug activation, laying the foundation for azide-masked compounds as a new class of ultrasound-responsive cancer therapeutics.

## Teaser

Low-intensity ultrasound enables aromatic azides to be reduced to amines through a single-electron transfer mechanism, enhanced by electron-withdrawing group, with co-loaded azide-masked resiquimod and riboflavin nanoparticles suppressing tumors.

## INTRODUCTION

Anti-tumor drugs often cause severe side effects because they act indiscriminately on both normal and tumor tissues [1–4]. Prodrugs have emerged as a promising strategy that employs stimuli-responsive chemical protecting groups to mask drug-active sites [5–8]. This approach allows prodrugs to remain dormant in normal tissues while selectively activated in tumor sites, thus significantly enhancing the therapeutic index [9,10]. Some prodrugs are designed to be activated by specific internal tumor environments, for example, tirapazamine [10] and EO9 (Apaziquone) [11,12]. However, due to tumor heterogeneity, the therapeutic effects of these prodrugs are often inconsistent, and their applicability is limited even for the same cancer type or different stages [13,14].

To overcome this limitation, external triggers can be employed to enable on-demand activation of prodrugs, thereby broadening the applicability of this technique to a larger patient population [15–18]. Ultrasound (US) is widely used for tumor diagnosis and therapy due to its real-time imaging capabilities, high efficiency, portability, and deep tissue penetration without harming healthy tissues [19–23]. The intensity of therapeutic US is significantly higher than that of diagnostic US [24–27]. In general, the intensity of diagnostic US is less than 0.1 W/cm^2^, while that of therapeutic US varies depending on the desired therapeutic effect [28,29]. High-intensity (>100 W/cm^2^) US can induce coagulative necrosis and tissue ablation [30–32]. For example, high-intensity focused US has been clinically used as a needle-free, non-ionizing thermal ablation technique for the treatment of uterine leiomyomas and other tumors [33–37]. Lower-intensity US, ranging from 0.125 to 3 W/cm^2^, can generate non-thermal effects via low-intensity mechanical energy, which transmits acoustic energy to the target tissue [38–40]. Clinically, low-intensity US is widely used to repair damaged tissue, relieve pain, and promote fracture healing [41,42]. Compared to light, heat and radiation, low-intensity US is a safe and effective external trigger [43–46], using its non-thermal effect to achieve selective activation of prodrugs. Through proper prodrug design, the selective activation of prodrugs via low-intensity US stimulation holds great potential for minimizing drug-related adverse effects [47–53]. This approach is expected to pioneer a new frontier in US therapy.

Herein, we present a new ultrasonic chemistry approach for the reduction of aromatic azides to their corresponding amines using low-intensity US in an aqueous solution. The presence of strong electron-withdrawing groups or low natural charges enhances the efficiency of this reduction reaction. Using azide-modified resiquimod (R848-N_3_) as a model prodrug, we clarified the mechanism of this aromatic azide reduction reaction, mediated by β-nicotinamide adenine dinucleotide disodium salt hydrate (NADH) via a single-electron transfer process. NADH provides the necessary hydrogen ion (H^+^) and electron, while riboflavin tetrabutyrate (TBR) acts as an electron transfer catalyst during the reduction process. By co-delivering R848-N_3_ and TBR co-loaded onto nanoparticles coupled with low-intensity US, precise tumor-selective reduction of R848-N_3_ was achieved *in vivo* with excellent immunotherapeutic effects without significant toxicity. These findings offer a solution promising to achieve on-demand activation of aromatic azide prodrugs *in vivo* using low-intensity US.

## RESULTS AND DISCUSSION

### Reduction of aromatic azide-containing compounds by low-intensity US

**Figure 1.**
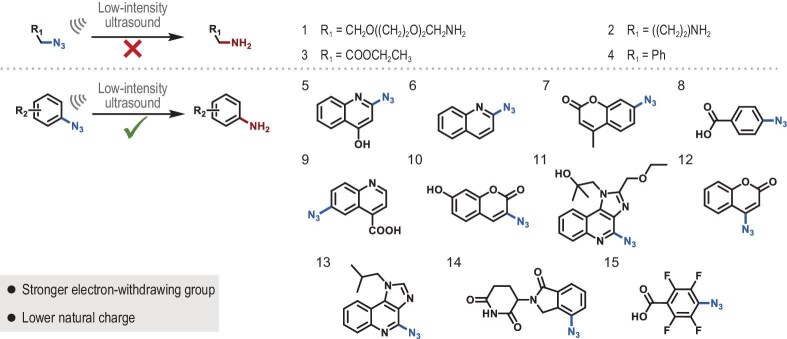
Ultrasonic reduction of azide-containing compounds.

Fifteen azides were prepared to test their reduction by low-intensity US (1.0 MHz, 50% duty cycle and 2.5 W/cm^2^, 5 min) in the presence of NADH and TBR (Fig. 1 and Figs S1–S9a). Aliphatic azides (**1–3**) and benzyl azide (**4**) were resistant to ultrasonic reduction, whereas aromatic azides were effectively reduced. Furthermore, azides with stronger electron-withdrawing groups or lower natural charges demonstrated enhanced reduction efficiency when their molecular structures are comparable (Table 1). For example, 2-azido-4-quinolinol (**5**) and 2-azidoquinoline (**6**) were reduced by 25.37% and 29.46%, respectively. Similarly, 7-azido-4-methylcoumarin (**7**) and azidobenzoic acid (**8**) were reduced by 37.81% and 43.19%, respectively, and 3-azido-7 hydroxycoumarin (**10**) (68.20%), R848-N_3_ (**11**) (69.40%), 4-azidocoumarin (**12**) (70.31%), and azide-modified imiquimod (**13**) (R837-N_3_, 70.41%) showed a higher reduction rate than 6-azidoquinoline-4-carboxylic acid (**9**) (48.27%). Lenalidomide-N_3_ (**14**) and 2,3,5,6-tetrafluoro-4-azidobenzoic acid (**15**) were reduced to corresponding amines under low-intensity US radiation at a reduction rate of 81.20% and 92.17%, respectively. Thus, low-intensity US can reduce aromatic azide-containing compounds to corresponding amines by using TBR as a catalyst, and the reduction rate is affected by the electron cloud distribution and conjugate structure. Our findings also indicate that aromatic azides containing stronger electron-withdrawing groups and lower natural charges exhibit a higher reduction rate.

**Table 1.** Ultrasonic reduction rates of azide-containing compounds and natural charge values of the chemical groups (R groups) to which the azide is attached. (US: 1.0 MHz, 50% duty cycle, 2.5 W/cm^2^, 5 min). The natural charge values of the R groups were determined by density functional theory.

Structure number	Reduction rate (%)	Natural charge
1	0.00	0.201
2	0.00	0.202
3	0.00	0.171
4	0.00	0.194
5	25.37	0.109
6	29.46	0.106
7	37.81	0.110
8	43.19	0.116
9	48.27	0.112
10	68.20	0.102
11	69.40	0.092
12	70.31	0.084
13	70.41	0.089
14	81.20	0.127
15	92.17	0.066

### The mechanism of US-mediated R848-N_3_ reduction

**Figure 2.**
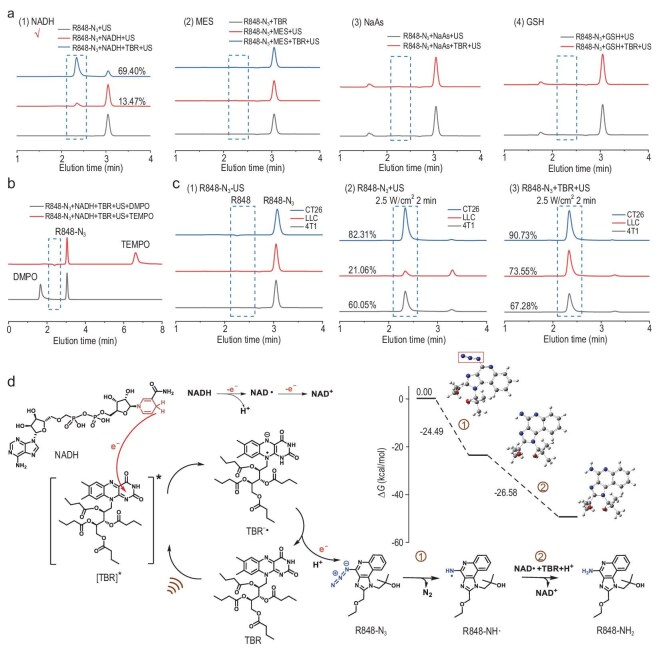
(a) HPLC curves of R848-N_3_ (1 mM, 1 equiv.) and riboflavin tetrabutyrate (TBR) (2 mM, 2 equiv.) in NADH, MES, NaAs or GSH (2 mM, 1 equiv.) under US conditions for 5 min. (b) In the presence of TEMPO or DMPO, HPLC curves of R848-N_3_ (1 mM, 1 equiv.), TBR (2 mM, 2 equiv.) and NADH (2 mM, 1 equiv.) after US for 5 min. (c) HPLC curves of R848-N_3_ or R848-N_3 _+ TBR in CT26, LLC and 4T1 tumor cell lines after US stimulation for 2 min. (d) The reduction mechanism of US-reduced R848-N_3_ and the values of Gibbs free energy (Δ*G*) calculated by density functional theory for the predicted reactions (B3LYP-D3(BJ)/def2-TZVP) at 1 atm and 298.15 K (US: 1.0 MHz, 50% duty cycle and 2.5 W/cm^2^).

To understand the mechanism of US-mediated reduction of aromatic azide-containing compounds, we further explored the effects of different reducing agents on reduction using R848-N_3_ as a model prodrug. R848-N_3_ and TBR were added to aqueous solutions containing NADH, 2-morpholinoethanesulfonic acid (MES), sodium ascorbate (NaAs) or glutathione (GSH) (Fig. 2a). Following US stimulation (1.0 MHz, 50% duty cycle and 2.5 W/cm^2^, 5 min), 69.40% of R848-N_3_ was reduced by NADH, while no reduction was observed in the solutions containing MES, NaAs or GSH. Moreover, TBR acted as an acoustic catalyst improving the US reduction rate of R848-N_3_ as deduced by the minimal reduction of R848-N_3_ (13.47%) upon removal of TBR. These findings suggest that NADH provides the necessary hydrogen ion (H^+^) and electron for the reduction of R848-N_3_, rather than these being obtained from water or other reducing agents. In addition, due to the slightly acidic characteristic of the tumor microenvironment, we further investigated the role of pH in the US-mediated reduction process. The results show that the reduction efficiency of R848-N_3_ with TBR is increased to 80.16% under acidic conditions (Fig. S9b), indicating that the addition of H^+^ ions in acidic environments may enhance the reaction, and the potential applicability of this reaction in biological systems where acidic conditions are prevalent.

The action of TBR as a catalyst for electron transfer during reduction was then investigated by determining whether TBR, like NADH, delivers electrons via a single-electron transfer process. Introduction of the radical scavengers 5,5-dimethyl-1-pyrroline-*N*-oxide (DMPO) or 2,2,6,6-tetramethylpiperidinooxy (TEMPO) was followed by the addition of R848-N_3_. R848-N_3_ was not reduced to R848 in this process, demonstrating that TBR is also converted to radicals (TBR^−^·) during electron transfer (Fig. 2b), reverting to the TBR form and continuing to participate in the reduction. Thus, TBR transfers electrons via single-electron transfer during US reduction.

US reduction of R848-N_3_ was studied under physiological conditions using CT26, LLC and 4T1 tumor cell lines, and the influence of the acoustic catalyst TBR was assessed. Without US stimulation, R848-N_3_ remained stable, with no unexpected R848 leakage. The reduction rate of R848-N_3_ in CT26, LLC and 4T1 tumor cell lines after US stimulation (1.0 MHz, 50% duty cycle and 2.5 W/cm^2^, 2 min) was 82.31%, 21.06% and 60.05%, respectively, and the reduction rate improved with the addition of TBR (CT26: 90.73%, LLC: 73.55% and 4T1: 67.28%) (Fig. 2c). Thus, under physiological conditions, US can induce the reduction of R848-N_3_ to R848 and TBR catalyzes the process.

Based on our findings, we suggest a potential reduction mechanism for R848-N_3_ induced by US (Fig. 2d). Under US stimulation, TBR is activated to an oxidant, [TBR]*, while NADH acts as a donor of H^+^ and electrons, producing two electrons, H^+^ and NAD^+^, in two steps. [TBR]* then obtains a single electron from NADH, resulting in the formation of the free radical TBR^−^·, which transfers electrons to R848-N_3_ through two single-electron transfer processes. With the participation of H^+^, this process activates R848-N_3_ to R848.

To validate this reduction process, density functional theory was employed to compute the predicted energy of the reaction (Δ*E*), the reaction enthalpy (Δ*H*), and the Gibbs free energy (Δ*G*) (Table S1). Because both reactions are exothermic, they can occur spontaneously under computational conditions, and the products of the first reaction can be formed. The first reaction has a lower Δ*H* than the second because the reactants in the first step are conventional closed-shell molecules (relatively stable), whereas the product contains two free radicals (relatively unstable). Nonetheless, in the case of entropy increase, Δ*H* <0 and Δ*G* <0, supporting the spontaneity of the reaction and confirming the existence of R848-NH·, likely due to the stability caused by the formation of N_2_, promoting the reaction. For the second reaction, as the reactants and products have one free radical each, the entropy changes before and after the reaction are insignificant, resulting in highly similar Δ*E*, Δ*H* and Δ*G* of the reaction, with differences of <1 kcal/mol. Thus, the final product R848-NH_2_ generated in this reaction is more stable than R848-NH· generated in the first step. The above results confirm the previous assumption that US can effectively reduce R848-N_3_ to R848, and the theoretical calculation validates the rationality of the reaction process, providing theoretical support for subsequent evaluation *in vivo*.

### Synthesis of NPs(R848-N_3 _+ TBR) and evaluation of US-mediated reduction *in vitro* and *in vivo*

**Figure 3.**
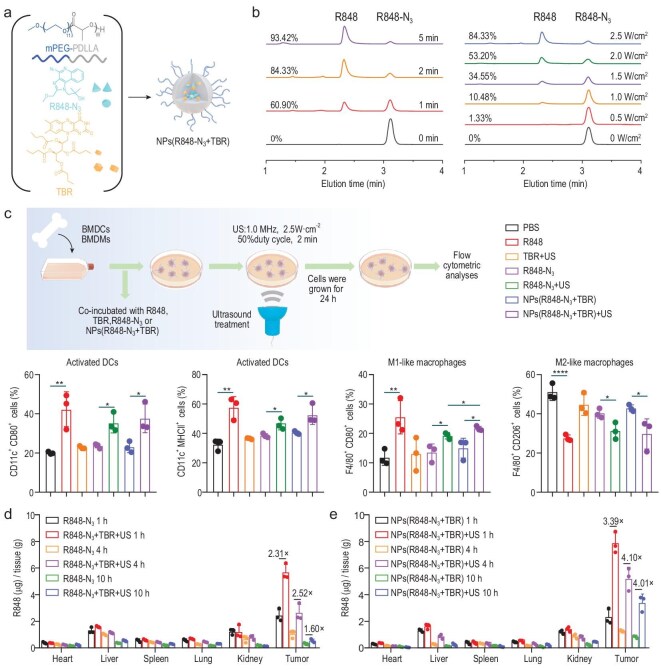
(a) Schematic diagram of NPs(R848-N_3_ + TBR) nanoparticles. (b) HPLC curves of NPs(R848-N_3_ + TBR) after US stimulation at a different power or time. (c) Illustration of the setup of US stimulation experiment *in vitro*. Flow cytometry was conducted to evaluate BMDCs activation and BMDMs polarization after different treatments (*n *= 3). The biodistribution of R848 in CT26 tumor-bearing mice (*n *= 3). Mice were treated with an i.v. administration of (d) R848-N_3_, R848-N_3_ + TBR or (e) NPs(R848-N_3_ + TBR) (20 mg·kg^-1^ R848-N_3_ and 72 mg/kg TBR) (US: 1.0 MHz, 50% duty cycle, 1.5 W/cm^2^ and 2 min). Results are expressed as mean ± SD (*n *= 3). *P* values were determined by one-way ANOVA and Tukey's test (*P < 0.05, **P < 0.01, ***P < 0.001 and ****P < 0.0001).

We further speculated that R848-N_3_ and TBR can be co-loaded onto nanoparticles (NPs) to improve their reduction efficacy. To test this, we co-loaded R848-N_3_ and TBR into mPEG-PDLLA NPs and obtained NPs(R848-N_3_ + TBR) (Fig. 3a). The structure and molecular weight (9.8 × 10^3^ g/mol) of mPEG-PDLLA were determined by ^1^H NMR (Fig. S10b). In addition, the drug loading content of R848-N_3_ and TBR can be determined by HPLC and UV–vis absorption spectrum as 9.53% and 34.33%, respectively. The results of dynamic light scattering (DLS) and transmission electron microscopy (TEM) indicate that NPs(R848-N_3_ + TBR) have appropriate size (115.9 nm) and negative charge (−5.78 ± 0.42 mV) (Fig. S10c, d). Under physiological conditions (pH 7.4), the NPs(R848-N_3_ + TBR) demonstrated minimal spontaneous drug release (<10% over 72 h), confirming their stability (Fig. S10e). However, upon US treatment (1.0 MHz, 50% duty cycle and 2.5 W/cm^2^, 2 min), the drug release increased to 72.08% in a slightly acidic environment (pH 5.5), demonstrating that NPs(R848-N_3_ + TBR) are expected to release effectively within tumors. Furthermore, the reduction rate of NPs(R848-N_3_ + TBR) reached 60.90% after 1 min and 84.33% after 2 min under low-intensity US radiation (1.0 MHz, 50% duty cycle and 2.5 W/cm^2^), which was positively correlated with the intensity of US (Fig. 3b). The viability of the CT26 and 3T3 cells after incubation with R848 or R848-N_3_ for 24 h was higher than 80% (Fig. S10f, g), suggesting undetectable cytotoxicity of the prodrug.

Given that R848 exhibits the capability to activate DCs and polarize macrophages, we further investigated the immunomodulatory impacts of R848-N_3_ and NPs(R848-N_3_ + TBR) on antigen-presenting cells under US conditions (1.0 MHz, 50% duty cycle and 1.5 W/cm^2^, 2 min). Following the different treatments of bone marrow-derived DCs (BMDCs), it was observed that the R848-N_3_ + US treatment significantly upregulated the proportion of activated DCs, which surpassed those recorded in the R848-N_3_ group. Similarly, NPs(R848-N_3_ + TBR)+US treatment also increased the proportion of DCs to 37.70% and 52.86% compared with the NPs(R848-N_3_ + TBR) alone (22.86% and 40.68%) (Fig. 3c and Fig. S11a). Importantly, US treatment at 1 W/cm² for 2 min did not significantly affect DC viability and the combination of US and R848-N_3_ treatment did not further decrease cell viability compared to US treatment alone, suggesting that the US parameters used in our study are safe for DCs (Fig. S11b). Flow analysis of the expression of markers in bone marrow-derived macrophages (BMDMs) following various treatments revealed a significant increase in M1-like macrophages post R848-N_3_ + US treatment (19.55% of all cells), compared with R848-N_3_ alone (13.68% of all cells). Conversely, there was a noticeable decrease in M2-like macrophages for the R848-N_3_ + US group (31.28% of all cells) and NPs(R848-N_3_ + TBR)+US group (29.80% of all cells), when compared to the PBS control group (51.17% of all cells) (Fig. 3c and Fig. S12). These findings suggest that R848-N_3_ and NPs(R848-N_3_ + TBR) can exert immunomodulatory effects under US conditions, and the increased number of activated DCs and M1-like macrophages is conducive to the elimination of tumors.

The biodistribution of R848-N_3_ or NPs(R848-N_3_ + TBR) in combination with low-intensity US (1.0 MHz, 1.5 W/cm^2^, 50% duty cycle, 2 min) was further investigated. The concentration of R848 within the tumor in the R848-N_3_ + US group was observed to be 2.52-fold compared to the tumor in the R848-N_3_ group at 4 h (Fig. 3d and Table S2, Table S3). In line with this, the tumor concentration of R848 in the NPs(R848-N_3_ + TBR)+US group was found to be 4.10-fold higher than that in the NPs(R848-N_3_ + TBR) group at 4 h (Fig. 3e and Table S4, Table S5). At 10 h, the NPs (R848-N_3_ + TBR)+US group showed a tumor-to-liver concentration ratio of 13.37 for R848. Thus, low-intensity US can achieve selective reduction of R848-N_3_ and NPs(R848-N_3_ + TBR) *in vivo*, and the activation of R848-N_3_ by US was significantly enhanced by co-loading R848-N_3_ and TBR into NPs. This is likely due to the co-delivery system increasing the collision probability between R848-N_3_ and TBR.

### NPs(R848-N_3_ + TBR) combined with low-intensity US for a selective therapy against tumors

**Figure 4.**
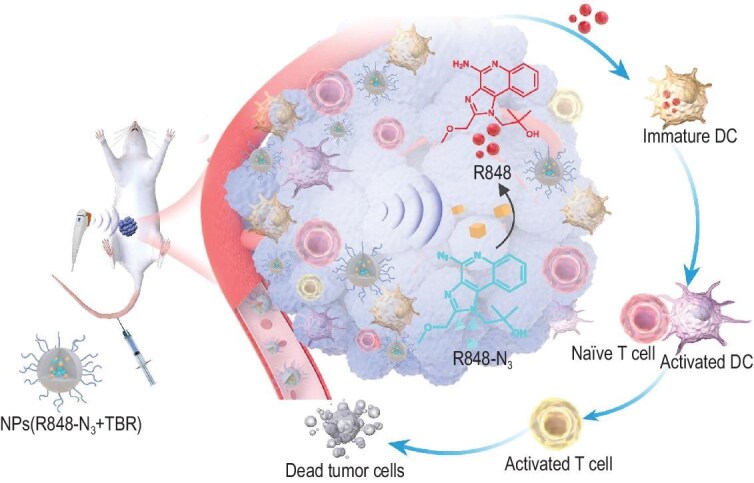
Schematic of NPs(R848-N_3_ + TBR)+US selectively activated by low-intensity ultrasound for tumor immune activation and growth inhibition. After intravenous administration, NPs(R848-N_3_ + TBR)+US reached the tumor site through blood circulation, and the R848-N_3_ prodrug was selectively reduced to R848 at the tumor site after low-intensity US stimulation. Then, R848 is internalized by immature DC to further activate DC. Finally, the activated DC primes T cells to kill tumor cells.

**Figure 5.**
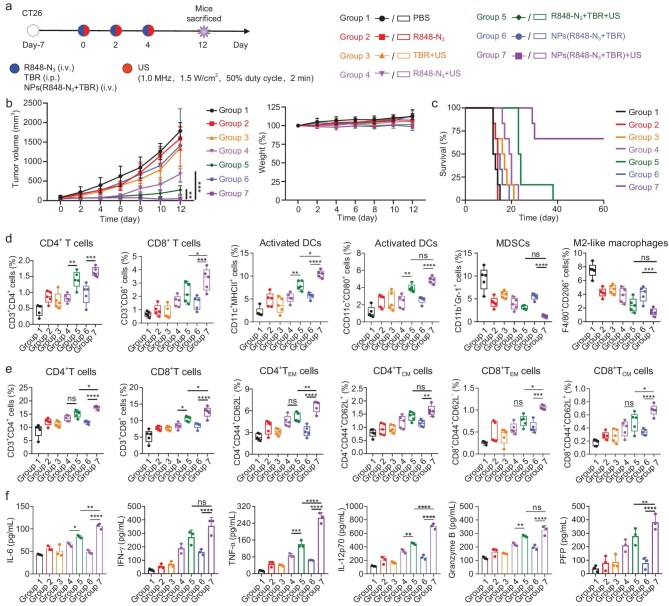
*In vivo* antitumor efficacy was evaluated in CT26 tumor-bearing mice. (a) Treatment regimen and specific groups of the subcutaneous CT26 tumor model. The mice were randomly allocated into seven groups: PBS, R848-N_3_, TBR + US, R848-N_3_ + US, R848-N_3_ + TBR + US, NPs(R848-N_3_ + TBR), NPs(R848-N_3_ + TBR)+US. Mice were administered with R848-N_3_ (20 mg/kg), TBR (72 mg/kg), NPs(R848-N_3_ + TBR) (210 mg/kg, based on R848-N_3_ and TBR) and US (1.0 MHz, 1.5 W/cm^2^, 50% duty cycle, 2 min) on day 0, 2, 4. (b) Tumor volume and the weight and (c) survival curves of CT26 tumor-bearing mice after receiving various treatments (*n *= 6). Flow cytometry results showing the percentage of different cell types (d) in CT26 tumors and (e) in spleen on day 12 after various treatments (*n *= 5). (f) Blood biochemical analysis of CT26 tumor-bearing mice treated with different formulations (*n *= 3). *P* values were determined by one-way ANOVA and Tukey's test (*P < 0.05, **P < 0.01, ***P < 0.001 and ****P < 0.0001).

The *in vivo* therapeutic and immune activation effects of NPs(R848-N_3_ + TBR) combined with low-intensity US (1.0 MHz, 1.5 W/cm^2^, 50% duty cycle, 2 min) were examined in CT26 tumor-bearing mice (Fig. 4). The treatment regimen and specific groups are shown in Fig. 5a. As expected, the tumor suppression rate (TSR) of the combination treatment R848-N_3_ + TBR + US reached 84.7%, while the TSR of free R848-N_3_ and R848-N_3_ + US was 9.9% and 61.9%, respectively. Of note, the TSR of NPs(R848-N_3_ + TBR)+US group was 99.0%, which was significantly greater than that for the NPs(R848-N_3_ + TBR) group (30.2%) (Fig. 5b). Consequently, the combined treatment demonstrated a more effective inhibition of tumor growth and near-complete eradication of the tumor by the conclusion of the observation period, without significantly affecting the body weight of mice (Fig. 5b). Furthermore, the survival rates of mice in different treatment groups were carefully monitored. These findings revealed that the mice in the NPs(R848-N_3_ + TBR)+US group maintained a notable survival rate (66.7%) on day 60 (Fig. 5c).

Given the potent antitumor effect of NPs(R848-N_3_ + TBR)+US, we further assessed its capacity to selectively stimulate immunity by observing the alterations within the tumor following treatments using flow cytometry (Fig. 5d). As anticipated, the NPs(R848-N_3_ + TBR)+US group exhibited a higher presence of CD3^+^CD4^+^ T cells (1.65% of all cells) within the tumor. In contrast, the TBR + US group and NPs(R848-N_3_ + TBR) group had CD3^+^CD4^+^ T cells at only 0.74% and 0.97% of all cells, respectively (Fig. S13). Similarly, there was a marked rise in CD3^+^CD8^+^ T cells (3.28% of all cells) in the NPs(R848-N_3_ + TBR)+US group compared to both the TBR + US group (0.81% of all cells) and NPs(R848-N_3_ + TBR) group (1.52% of all cells) (Fig. S13). Furthermore, an increased number of activated DCs (10.46% of all cells) was observed within the tumor for the NPs(R848-N_3_ + TBR)+US group, as evidenced by the CD11c^+^MHCII^+^. This was markedly greater than that in the TBR + US group and the NPs(R848-N_3_ + TBR) group, with the ratio of activated DCs being 2.85-fold and 1.85-fold greater, respectively (Fig. S14). Similar trends were observed for activated DCs identified by the CD80^+^ population in the CD11c^+^ population. These findings indicate that the combined treatment markedly improves tumor immunity and bolsters antitumor efficacy. Furthermore, the proportion of myeloid-derived suppressor cells (MDSCs) in the NPs(R848-N_3_ + TBR)+US group was 1.24%, compared to both the TBR + US group (5.95%) and NPs(R848-N_3_ + TBR) group (5.49%) (Fig. S15). Only 1.42% of all cells in the NPs(R848-N_3_ + TBR)+US group were identified as M2-like macrophages, whereas a significantly higher presence of these macrophages was observed in the US group (4.73% of all cells) and the NPs(R848-N_3_ + TBR) group (4.06% of all cells) (Fig. S15). The examination of immune cells within the tumor revealed that R848 produced by US reduction effectively reversed the highly immunosuppressive tumor microenvironment.

To assess systemic immune reactions, spleen-derived immune cells were subjected to flow cytometry analysis. The findings revealed that the percentage of CD3^+^CD8^+^ T cells in the NPs(R848-N_3_ + TBR)+US group was 12.64%, which surpassed that of the TBR + US group (7.73%) and the NPs(R848-N_3_ + TBR) group (8.58%) (Fig. 5e and Fig. S16). Compared to other groups, the NPs(R848-N_3_ + TBR)+US group exhibited the strongest systemic immune response and the highest number of central memory T (T_CM_) cells and effector memory T (T_EM_) cells, indicating that combination treatment can stimulate immune memory effects (Fig. 5e and Fig. S16). Additionally, the combined treatment resulted in upregulation of six pro-inflammatory cytokines in the serum (Fig. 5f). These findings suggest that NPs(R848-N_3_ + TBR)+US therapy can effectively activate both the immune response and systemic immune response to achieve accurate immunotherapy of tumors.

To further estimate the therapeutic effects, the excised tumors were processed for hematoxylin and eosin (H&E) analyses (Fig. S17). The findings indicate that the tumor has been largely necrotic and almost completely eliminated after treatment with NPs(R848-N_3_ + TBR)+US. Furthermore, blood chemistry assessments revealed no notable alterations, suggesting that the combined treatment did not exert a substantial influence on liver and kidney function (Fig. S18).

## CONCLUSION

Taken together, we found a new ultrasonic chemistry reaction, in which aromatic azides are reduced to the corresponding amines through low-intensity US. The reduction mechanism occurs via a single-electron transfer process, with TBR serving as a catalyst to improve the reduction rate. Aromatic azides with stronger electron-withdrawing groups or lower natural charge exhibit higher reduction efficiency. When R848-N_3_ and TBR co-loaded nanoparticles were used in parallel with low-strength US, the concentration of R848 within the tumor was found to be 13.37-fold higher than in the liver, demonstrating superior tumor selectivity compared to small-molecule prodrugs. Due to its excellent tumor selectivity, the NPs(R848-N_3_ + TBR)+US group exerted remarkable therapeutic efficacy. It can significantly improve the tumor immunosuppressive microenvironment and induce a systemic immune response, with a tumor suppression rate of 99.0%. Therefore, as a portable and safe therapeutic modality with high penetration depth, low-intensity US can activate prodrugs *in vivo* on demand to achieve selective tumor therapy through systemic administration.

## ETHICAL APPROVAL DECLARATION

Female BALB/c mice, aged between 6 and 8 wk with an average body weight of 19–20 g, were procured from Beijing Vital River Laboratory Animal Technology Co. Ltd. (Beijing, China). For the generation of the CT26 colon cancer model, CT26 cells (1.0 × 10^6^ per mouse) were subcutaneously injected into the right flank of these mice. All animal studies adhered to guidelines approved by the Animal Welfare and Ethics Committee of Changchun Institute of Applied Chemistry, Chinese Academy of Sciences.

## Supplementary Material

nwaf140_Supplemental_File
