# Supplementary material for: Ultrasound-induced single-electron reduction of azide groups in aromatic prodrugs
Source: Natl Sci Rev. 2025 Apr 10;12(6):nwaf140. doi: 10.1093/nsr/nwaf140 (PMC12086671; doi:10.1093/nsr/nwaf140)
Supplement: nwaf140_Supplemental_File [file nwaf140_supplemental_file.pdf]

Ultrasound-Induced Single-Electron Reduction of Azide Groups in Aromatic Prodrugs

Jiali Sun[1,2,3], Haochen Yao[4,*], Chenguang Yang[1], Fuxin Xue[5], Xitong Ren[1,2], Linjie Cui[1,2], Zhilin Liu[1,*], Zhaohui Tang[1,2,*] and Xuesi Chen[1,2]

[1] Dr. J. Sun, C. Yang, X. Ren, L. Cui, Dr. Z. Liu, Prof. Z. Tang and Prof. X. Chen

Key Laboratory of Polymer Ecomaterials

Changchun Institute of Applied Chemistry, Chinese Academy of Sciences,

Changchun 130022, China

E-mail: ztang@ciac.ac.cn; zlliu@ciac.ac.cn

[2] Dr. J. Sun, X. Ren, L. Cui, Prof. Z. Tang and Prof. X. Chen

School of Applied Chemistry and Engineering

University of Science and Technology of China

Hefei 230026, China

[3] Dr. J. Sun

Key Laboratory of Zoonosis Research, Ministry of Education,

College of Basic Medical Science, Jilin University

Changchun 130021, China

[4] Dr. H. Yao

Hepatobiliary and Pancreatic Surgery Department, General Surgery Center,

First Hospital of Jilin University,

Changchun 130021, China

E-mail: yaohaochen@jlu.edu.cn

[5] Dr. F. Xue

Department of Radiation Oncology,

China-Japan Union Hospital of Jilin University,

Changchun 130033, China

*Corresponding authors. E-mails: ztang@ciac.ac.cn; zlliu@ciac.ac.cn; yaohaochen@jlu.edu.cn

Ethical Approval Declaration

Female BALB/c mice, aged between 6–8 weeks with an average body weight of 19–20 g, were procured from Beijing Vital River Laboratory Animal Technology Co. Ltd. (Beijing, China). For the generation of the CT26 colon cancer model, CT26 cells (1.0×10^6 per mouse) were subcutaneously injected into the right flank of these mice. All animal studies adhered to guidelines approved by the Animal Welfare and Ethics Committee of Changchun Institute of Applied Chemistry, Chinese Academy of Sciences.

Experimental Procedures

Materials.

Benzyl azide, azidobenzoic acid, and 2,3,5,6-tetrafluoro-4-azidobenzoic acid were procured from Shanghai Jiuding Chemical Co., Ltd. (China). 3-Azido-1-propylamine, riboflavin tetrabutyrate (TBR), 4-acetamidobenzenesulfonyl azide, 3-azido-7-hydroxycoumarin, and 7-amine-4-methylcoumarin were obtained from Shanghai Macklin Biochemical Technology Co., Ltd. (China). Azidoacetic acid ethyl ester and imiquimod were purchased from Energy Chemical (China). 2-aminequinoline, 2-amine-4-quinolinol, 4-azidocoumarin, lenalidomide, 5,5-dimethyl-1-pyrroline-N-oxide (DMPO) and 2,2,6,6-tetramethylpiperidinooxy (TEMPO) were acquired from Aladdin Reagent (Shanghai) Co., Ltd. (China). 11-Azido-3,6,9-trioxaundecylamine, 2,4,6-triisopropylbenzene-sulfonyl azide, and 6-aminequinoline-4-carboxylic acid were procured from Shanghai Leyan Co., Ltd. (China). Resiquimod (R848) was sourced from Nanjing Sunsure Chemical Technology Co., Ltd., China. The diazotizing species fluorosulfuryl azide (FSO_2N_3) was prepared as described in a previous report.[1] Monomethoxy polyethylene glycol ($mPEG_{5k}$, average molecular weight = 5.0×10^3 g/mol) and 3-(4,5-Dimethylthiazol-2-yl)-2,5-diphenyl tetrazolium bromide (MTT) were obtained from Sigma-Aldrich (Beijing, China). RPMI 1640 and PBS buffer were obtained from Dalian Meilun Biotechnology Co., Ltd. (China). Fetal bovine serum (FBS) was procured from Zhejiang Tianhang Biotechnology Co., Ltd., China. Penicillin and streptomycin were sourced from Huabei Pharmaceutical Co. Ltd., China. The

antibodies used for flow staining were sourced from Biolegend. All other reagents and solvents utilized in this study were procured from Sinopharm Chemical Reagent Co., Ltd., China.

Characterization

The ^1H NMR spectrum was obtained using a Bruker AV 300 NMR spectrometer, operated in DMSO-d_6 at a temperature of 25 ℃. The chemical shifts were expressed in parts per million relative to tetramethylsilane (TMS). Electrospray ionization mass spectroscopy (ESI-MS) was executed utilizing a Bruker APEX-IV Fourier transform mass spectrometer. UV-Vis absorption spectra were meticulously recorded using a Lambda 365 UV-Vis spectrophotometer, sourced from PerkinElmer, USA. Dynamic laser scattering (DLS) measurements were conducted employing a Malvern zetasizer instrument, specifically the Nano-ZS90 model. High-performance liquid chromatography (HPLC) was performed on a Waters 2414 Refractive Index Detector, complemented by a Waters 515 HPLC pump and a C-18 column, both of which are part of the Symmetry® series. The acquisition of UV-vis absorption spectra was achieved through the utilization of a UV-vis spectrophotometer, specifically the 2401PC model from Varian. Ultrasonic (US) irradiation was administered using a DJO-2776 sonicator.

General Procedure: Synthesis and Characterization of Azide-Containing Compounds

To synthesize the azide-containing compounds, a solution of amine-containing compounds (1.0 mmol, in 5 mL DMF) was added to a 50-mL glass flask. Subsequently, a solution of FSO_2N_3 (comprising 1.0 mmol FSO_2N_3 and 200 mM in DMF/methyl tert-butyl ether (MTBE) at a ratio of 1:1, roughly 5 mL) along with aqueous $KHCO_3$ (3.0 M, 1.33 mL) was added. The diazotizing agent, fluorosulfuryl azide (FSO_2N_3), was synthesized as outlined in a prior study [1]. The mixture underwent stirring at 30 ℃ for a duration of 5 hours. Upon completion, ethyl acetate (40 mL) was introduced, followed by sequential washing of the resultant mixture using saturated brine. Subsequent drying was performed over Na_2SO_4, followed by concentration via rotary evaporation and vacuum drying. Crude products were further

refined through silica gel chromatography employing CH_2Cl_2/MeOH as the solvent at a ratio of 100:3, yielding the desired azide-containing compounds.

Computational Studies

The reduction mechanism of US-reduced R848-N_3 was investigated using density functional theory (DFT) with the B3LYP-D3(BJ) functional and the def2-TZVP basis set. All calculations were performed under standard conditions (1 atm and 298.15 K) using the Gaussian 16 software package. The geometries of all reactants, transition states, and products were fully optimized, and frequency calculations were performed to confirm the absence of imaginary frequencies (for minima) or the presence of a single imaginary frequency (for transition states). The Gibbs free energy (ΔG) values were calculated based on the optimized geometries and frequency analysis results.

Natural charges were calculated using Natural Bond Orbital (NBO) analysis to evaluate electron distribution and charge transfer during the reaction. The NBO analysis was performed using the NBO 6.0 module implemented in Gaussian 16, at the same level of theory (B3LYP-D3(BJ)/def2-TZVP) as the geometry optimization and energy calculations. The natural charges provided detailed insights into the electron redistribution and charge transfer mechanisms during the reduction process.

Synthesis and Characterization of mPEG$_{5k}$-PDLLA$_{5k}$

The diblock copolymer of mPEG$_{5k}$-PDLLA$_{5k}$ was synthesized via ring-opening polymerization, employing a 1:1 monomer mass ratio [2]. Firstly, mPEG$_{5k}$ (10.0 g, 2.0 mmol) was subjected after drying using toluene. Next, *D*, *L*-lactide underwent purification through recrystallization using ethyl acetate. To this process, 10.0 g (64.4 mmol) of *D*, *L*-lactide and 20.0 mg (0.05 mmol) of $Sn(Oct)_2$ were introduced into the flask containing mPEG$_{5k}$. The reaction was carried out in a nitrogen atmosphere at a temperature of 125 ℃ for 24 h. The resulting mPEG$_{5k}$-PDLLA$_{5k}$ copolymer was then purified by precipitation in cooled diethyl ether. Characterization of the block copolymer was achieved through ^{1}H NMR. Specifically, for mPEG$_{5k}$-PDLLA$_{5k}$, the signal corresponding to -OCH_2CH_2- (a+b) was observed at δ 3.64 ppm, while the signals for $CH(CH_3)$- (c) and -CH_3- (d) in the PDLLA$_{5k}$ unit were observed at δ 1.47–

1.61 ppm and δ 5.10–5.25 ppm, respectively. The molecular weight of mPEG$_{5k}$-PDLLA$_{5k}$, as determined by ^{1}H NMR, was found to be 9.8×10^3 g/mol.

Preparation of NPs(R848-N$_3$+TBR)

NPs(R848-N$_3$+TBR) were synthesized using the thin-film hydration technique. Initially, the drugs and mPEG$_{5k}$-PDLLA$_{5k}$ were jointly dissolved in CH_2Cl_2 at a mass ratio of R848-N$_3$ and TBR to mPEG$_{5k}$-PDLLA$_{5k}$ of 10% and 40%, respectively. This solvent was then evaporated under controlled conditions using a rotary evaporator. Following this, the mixture was reintroduced into deionized water, leading to the formation of micelles via self-assembly. These micelles were subsequently filtered through a 0.22-μm membrane to eliminate unincorporated drug aggregates prior to lyophilization. The drug loading content (DLC) values of R848-N$_3$ and TBR were measured as 9.53% and 34.33%, respectively. The DLC value of R848-N$_3$ was determined using a UV-HPLC system consisting of a C-18 column and a UV-vis detector at λ_{ex}=210 nm, with an injection volume of 20 μL and a mobile phase acetonitrile and water in a ratio of 80:20 (v/v), with a flow rate at 1.0 mL/min. The DLC value of TBR was calculated by UV–vis absorption spectrum (λ=443 nm). The resulting nanoparticles had a hydrodynamic diameter of approximately 115.9 nm in PBS (pH 7.4). The zeta potential value is -5.78 ± 0.42 mV.

Drug Reduction in Vitro

The azide-containing compounds (20.0 mM) were dissolved in DMSO and diluted in PBS (pH 7.4) before reaction with NADH (2.0 mM, 1.0 equiv.) and TBR (2.0 mM, 2.0 equiv.). Samples were degassed with nitrogen prior to the reaction and sealed in separate tubes. US (1.0 MHz, 50% duty cycle and 2.5 W/cm^2, 5 min) was then performed at room temperature. Each reaction was terminated with acetonitrile and the concentrations of azide-containing and amine-containing compounds were detected by UV spectrophotometer or HPLC eluted with acetonitrile:H_2O (65:35, v/v) and 0.05% (vt%) acetate acid, at a flow rate of 1.0 mL/min and detected at 210 nm. The recovery and reduction rates were calculated as follows: Reduction Rates = C_{amine} / $C_{initial}$, where $C_{initial}$ = 1.0 mM.

To better understand the mechanism of US reduction of aromatic azide-containing compounds, R848-N_3 (1.0 mM) and TBR (2.0 mM, 2 equiv.) were added to an aqueous solution containing NADH, 2-morpholinoethanesulfonic acid (MES), sodium ascorbate (NaAs), or glutathione (GSH) (2.0 mM, 1.0 equiv.) for US detection (1.0 MHz, 50% duty cycle and 2.5 W/cm^2, 5 min). Under the same conditions, radical scavengers DMPO (1.0 mM, 1.0 equiv.) or TEMPO (1.0 mM, 1.0 equiv.) were added to verify whether US reduction of azide was a single electron transfer process. To further understand the effect of pH on the ultrasonic reduction process, R848-N_3 (1.0 mM) and TBR (2.0 mM, 2 equiv.) were added to a solution with pH=5.5 for US detection (1.0 MHz, 50% duty cycle, 2.5 W/cm^2, 5 min).

The bioreduction potential of R848-N_3 was assessed in three distinct tumor cell types (CT26, 4T1, LLC) to ascertain the reduction capability of R848-N_3 under US exposure. The cells were exposed to a combination of R848-N_3 (5.0 μg/mL) and TBR (2.0 mM, 2.0 equiv.) under US conditions (1.0 MHz, 50% duty cycle and 2.5 W/cm^2, 2 min), and then analyzed by HPLC eluted with an acetonitrile and H_2O solution (65:35, v/v) and 0.05% (vt%) acetate acid, and detected at 210 nm, operating at a flow rate of 1.0 mL/min.

The reduction of NPs(R848-N_3+TBR) after US stimulation at different intensity or time was determined by HPLC. NPs(R848-N_3+TBR (3.57 mg/mL; based on 1 mM R848-N_3) were mixed with 2 mM NADH for US detection (1.0 MHz, 50% duty cycle and 2.5 W/cm^2) at different times (0 min, 1 min, 2 min, 5 min). The reduction of NPs(R848-N_3+TBR) at different US intensities (0, 0.5, 1.0, 1.5, 2.0, and 2.5 W/cm^2) was also detected at a set US time (2 min).

Cell Lines

Cells from the CT26 murine colon carcinoma cell line were cultured in RPMI-1640 (containing 10% FBS, 100 U/mL penicillin and 100 μg/mL streptomycin) at 37 ℃ in an atmosphere of 5% CO_2.

Stability detection of NPs(R848-N_3+TBR)

NPs(R848-N_3+TBR) (10 mg, dissolved in 50 mL PBS) was added to dialysis bag (MW 3500 Da), and shaken (80 rpm) at 37 °C in buffer solution (PBS, pH = 7.4) or slightly acidic environment (pH = 5.5). The ultrasonic conditions in a slightly acidic environment are: 1.0 MHz, 50% duty cycle and 2.5 W/cm^2, 2 min. At each time point, 5.0 mL of the release solution was withdrawn and 5.0 mL of the same fresh buffer was replenished. The release tests were repeated in triplicate under the same conditions. The amount of released R848-N_3 was measured by HPLC, with a mixture of acetonitrile:H_2O (65:35, v/v, including 0.05% acetic acid) and a flow rate of 1.0 mL/min.

In vitro cytotoxicity assay

The in vitro cytotoxicities of R848 and R848-N_3 were evaluated using MTT assay. 8000 CT26 cells or 3T3 cells were seeded per well with 200 µL of complete culture medium to 96-well culture plates. After overnight incubation, different concentrations of R848 or R848-N_3 was added. MTT assay test was carried out after incubation for 24h. The absorbances of each well were measured at 490 nm on a Bio-Rad 680 microplate reader. The relative cell viability (%) was determined through comparing the absorbance values of sample wells with that of control wells.

In Vitro Activation of DCs and Macrophage Polarization in Response to R848-N_3/NPs(R848-N_3+TBR) under US

Bone marrow-derived dendritic cells (BMDCs) and bone marrow-derived macrophages (BMDMs) were obtained from the femurs and tibias of 4–5-week-old BALB/c mice, respectively. BMDCs were generated by stimulating cells with IL-4 (10 ng/mL, Peprotech) and GM-CSF (20 ng/mL, Peprotech) for 7 days, while macrophages were generated by stimulating cells with M-CSF (80 ng/mL, Peprotech) for 7 days. Subsequently, 3×10^5 BMDCs and BMDMs were cultured in 2.0 mL of RPMI 1640 and 5 µg/mL of R848 (dissolved in the mixed solution of ethanol: Cremophor EL®:PBS (15:15:70, v/v/v)), 5 µg mL^{-1} of R848-N_3 (dissolved in a mixed solution of ethanol: Cremophor EL®:PBS (15:15:70, v/v/v)), NPs(R848-N_3+TBR) (including 5 µg/mL of R848-N_3, dissolved in PBS), TBR (20 µg/mL, dissolved in a

mixed solution of DMSO, ethanol, Cremophor EL® and PBS (5:25:25:45, v/v/v/v)), or a control from different groups for 12 h. The cells were then treated with US (1.0 MHz, 50% duty cycle and 1.5 W/cm^2, 2 min). BMDCs were collected and labled with CD11c, CD80 and CD86 antibodies. BMDMs were labled with F4/80, CD206 and CD80 antibodies. The activation of dendritic cells (DCs) and macrophage polarization were examined using flow cytometry.

In addition, we evaluated the effect of US treatment on cell viability using MTT assay. 8000 DCs were seeded per well with 200 μL of complete culture medium to 96-well culture plates. After overnight incubation, R848-N_3 was added. The cells were then treated with US (1.0 MHz, 50% duty cycle and 1.5 W/cm^2) for 2 min or 10 min. MTT assay test was carried out after incubation for 24h. The absorbances of each well were measured at 490 nm on a Bio-Rad 680 microplate reader. The relative cell viability (%) was determined through comparing the absorbance values of sample wells with that of control wells.

Biodistribution Study

To establish CT26 tumor models, female BALB/c mice aged 6–8 weeks were intraperitoneally injected with 1.0×10^6 CT26 cells per mouse. R848-N_3 was formulated in a solution of ethanol:Cremophor EL®:PBS (15:15:70, v/v/v). TBR was prepared in a mixed solution of DMSO:ethanol:Cremophor EL®:PBS (5:25:25:45, v/v/v/v), while NPs(R848-N_3+TBR) were dissolved in PBS. Upon reaching an approximate tumor volume of 300 mm^3, the mice were administered with R848-N_3 (20 mg kg^{-1}), R848-N_3+TBR (20 mg kg^{-1} R848-N_3 and 72 mg kg^{-1} TBR), and NPs(R848-N_3+TBR) (based on 20 mg kg^{-1} R848-N_3 and 72 mg kg^{-1} TBR). Four hours post-injection, the mice underwent US treatment (1.0 MHz, 1.5 W/cm^2, 50% duty cycle, 2 min). At 1, 4, and 10 h after treatment, the mice were euthanized, and their tumors and organs were harvested, accurately weighed, and subsequently ground into fragments. The tissues were then homogenized with a mixture of acetonitrile:H_2O (65:35, v/v, including 0.05% acetic acid) at a final concentration of 100 mg mL^{-1}. Following centrifugation at 1000×*g* for 20 min at 4 °C, the supernatant was analyzed

by HPLC. The eluate was then extracted with the acetonitrile:H_2O solution and detected at a wavelength of 210 nm at a flow rate of 1.0 mL/min.

In Vivo Antitumor Efficacy

The subcutaneous CT26 tumor model was generated by administering 1.0×10^6 CT26 cells into the right flank of female BALB/c mice, aged between 6 and 8 weeks. Upon reaching a tumor volume of approximately 70 mm^3, these mice were randomly segregated into seven distinct groups (*n*=6 each): PBS (Group 1), R848-N_3 (Group 2), TBR+US (Group 3), R848-N_3+US (Group 4), R848-N_3+TBR+US (Group 5), NPs(R848-N_3+TBR) (Group 6), NPs(R848-N_3+TBR)+US (Group 7). Mice were treated with R848-N_3 (20 mg kg^{-1}, i.v.), TBR (72 mg kg^{-1}, i.p.), NPs(R848-N_3+TBR) (210 mg kg^{-1}, i.v.) or US (1.0 MHz, 1.5 W/cm^2, 50% duty cycle, 2 min) on days 0, 2, and 4. Tumor volume was measured every other day using the Vernier calipers to assess the antitumor effect, and the body weight was measured to evaluate systemic toxicity. Tumor volume was calculated as $V = a \times b^2/2$, where a is the major axis and b is the minor axis of the tumor. The tumor suppression rate (TSR) was calculated using the formula TSR (%) = $[(V_c - V_x)/V_c] \times 100\%$, where V_c and V_x are the mean tumor volume of the PBS and the treatment groups, respectively. Upon the conclusion of treatment, the mice were sacrificed by inhaling isoflurane and tumors and primary organs from each group were harvested. These were subsequently fixed in a 4% (w/v) PBS-buffered paraformaldehyde solution for a duration of 48 hours, before being embedded in paraffin. The paraffin-embedded tumors and organs were then sectioned into 5-μm-thick slices and stained using hematoxylin and eosin.

Flow Cytometry Analysis

CT26 cells (1.0×10^6) were subcutaneously administered into the abdomens of female BALB/c mice, aged between 6 and 8 weeks. Upon reaching a tumor volume of approximately 70 mm^3, these mice were randomly segregated into ten groups, each comprising five individuals, and subjected to identical treatment protocols as previously delineated. Mice were euthanized on day 12, after which a single cell suspension of the tumor was procured through grinding. Spleen tissue samples underwent mechanical disintegration and resuspension in RPMI 1640, followed by

filtration, centrifugation, additional filtration, and red blood cell lysis. This process yielded a single cell suspension, which was subsequently incubated with an array of antibodies targeting immune cells. Flow cytometry analysis was executed using a BD FACS Canto II flow cytometer, with results subsequently analyzed via FlowJo software. The cells were labled with antibodies (CD3 (Catalog No. 100204), CD8a (Catalog No. 100711) and CD4 (Catalog No. 100422) antibodies for T cells; CD11c (Catalog No. 117308), CD80 (Catalog No. 104713) and MHC II (Catalog No. 107627) antibodies for activated DCs; CD11b (Catalog No. 101225) and Gr-1 (Catalog No. 108408) antibodies for MDSCs; F4/80 (Catalog No. 123114) and CD206 (Catalog No. 141704) antibodies for M2-like macrophages, F4/80 and CD80 antibodies for M1-like macrophages; CD3, CD8a, CD4, CD62L (Catalog No. 104428) and CD44 (Catalog No. 103007) antibodies for T cells and memory T cells). All these antibodies were purchased from BioLegend, Inc. The percentage reflects the ratio of specific immune cells to the total live population.

Inflammatory Cytokine Analysis

Samples of whole blood were collected on day 12 after therapy to analyze inflammatory cytokines (n=3). The serum was isolated from the clotted blood through centrifugation, conducted at 1000 $\times g$ and maintained at a temperature of 4 ℃ for a duration of 20 minutes. Subsequent analysis of the serum levels of interleukin 6 (IL-6), interferon-γ (IFN-γ), tumor necrosis factor-α (TNF-α), interleukin 12(IL-12p70), granzyme B and granzyme (PFP) were performed using an enzyme-linked immunosorbent assay (ELISA). This procedure adhered strictly to the manufacturer's guidelines.

Furthermore, biochemical assessments were conducted to assess liver and kidney functionality. This was accomplished by quantifying the serum concentrations of aspartate aminotransferase (AST), alanine aminotransferase (ALT), blood urea nitrogen (BUN), and creatinine (CRE).

Statistical Analysis

All experiments were conducted at least three times, with the results expressed as means ± standard deviation (s. d.). Comparisons between two groups were executed

using a two-tailed unpaired Student's *t*-test, while comparisons across multiple groups were facilitated by one-way ANOVA, supplemented by the corresponding Tukey's multiple comparison test.

Synthesis of Compound 5

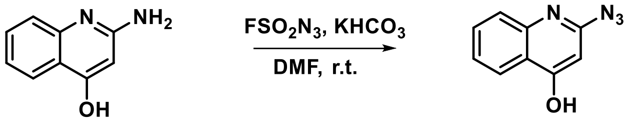

5

2-Azido-4-quinolinol was synthesized following a general procedure obtaining an 85% yield.

^{1}H NMR (**5**, 300 MHz, DMSO-d$_6$): δ_H 7.24 (1H, *d*, H-d), 7.56 (1H, *d*, H-e), 7.67 (1H, *d*, H-c), 7.95 (1H, *s*, H-a), 8.24 (1H, *d*, H-b). ESI-MS (*m/z*): calculated for $C_9H_6N_4O$ $[M+H]^+$ 187.2, found 187.2.

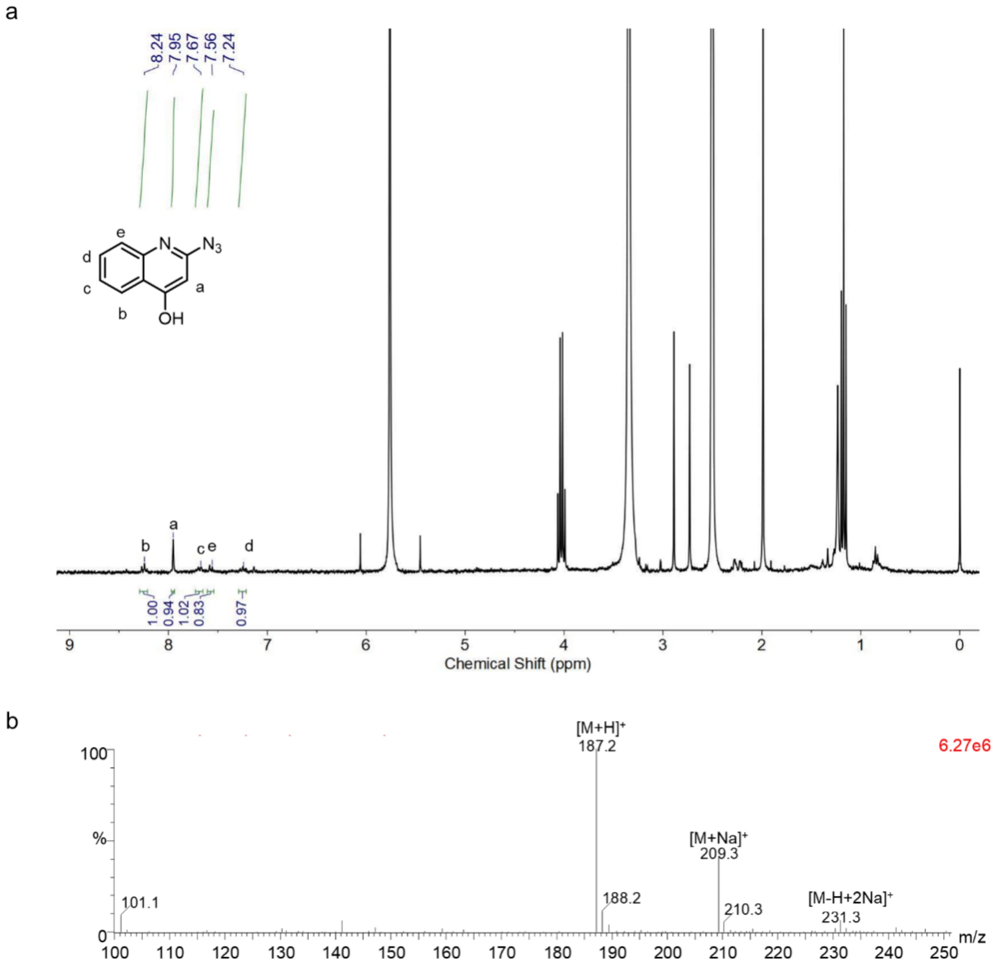

Figure S1. (a) ^{1}H NMR spectrum of 2-azido-4-quinolinol in DMSO-d$_6$. (b) ESI-MS (ESI$^+$) spectrum of 2-azido-4-quinolinol.

Synthesis of Compound 6

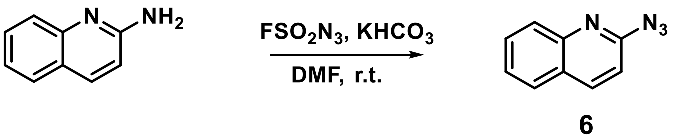

The synthesis of 2-azidoquinoline was executed in accordance with a standard procedure, yielding an impressive 78%.

^{1}H NMR (**6**, 300 MHz, DMSO-d$_6$): δ_H 7.84 (*m*, 1H, H-a), 8.00 (*m*, 2H, H-d, H-e), 8.12 (*d*, 1H, H-f), 8.30 (*d*, 1H, H-c), 8.67 (*d*, 1H, H-b). ESI-MS (*m/z*): calculated for $C_9H_6N_4$ $[M+H]^+$ 171.2, found 171.2.

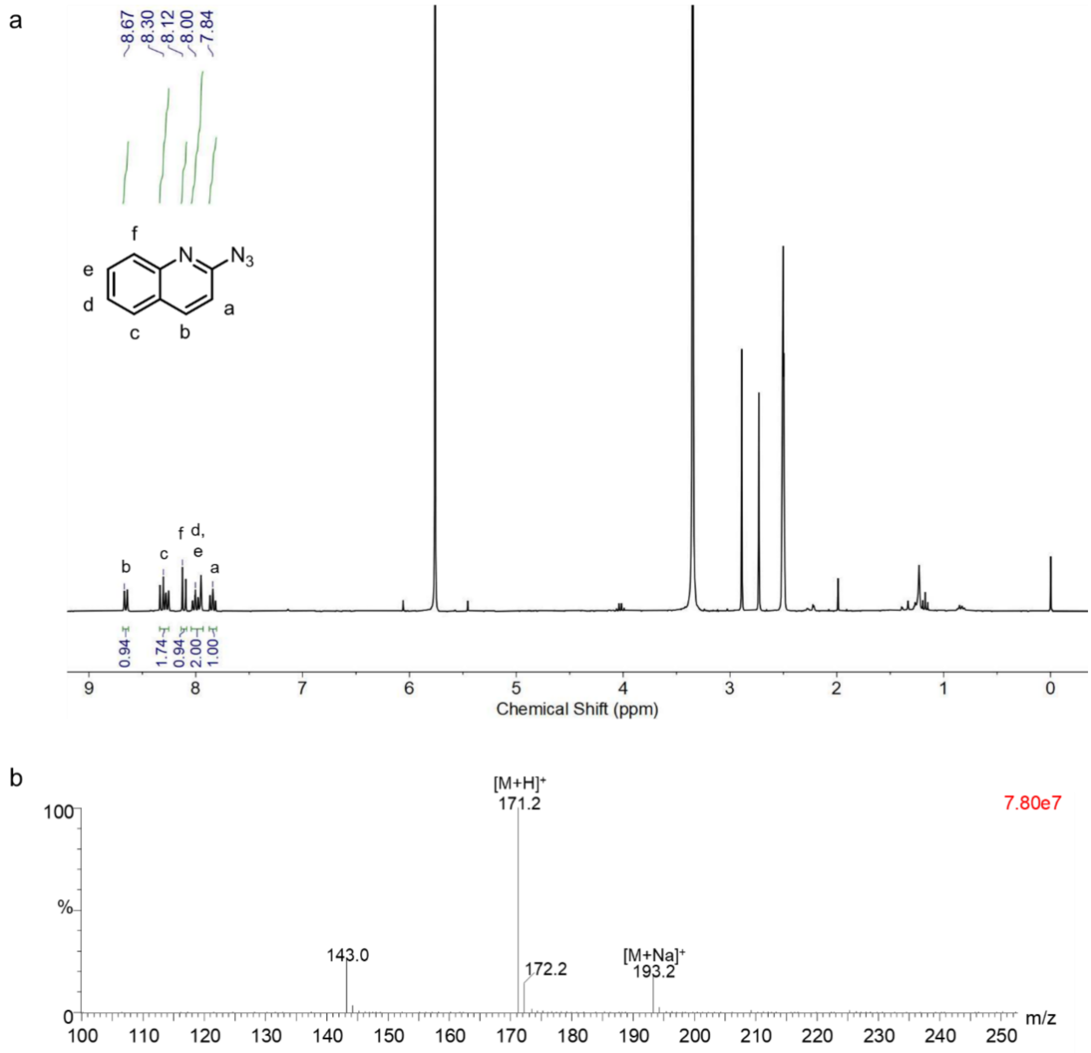

Figure S2. (a) ^{1}H NMR spectrum of 2-azidoquinoline in DMSO-d$_6$. (b) ESI-MS (ESI$^+$) spectrum of 2-azidoquinoline.

Synthesis of Compound 7

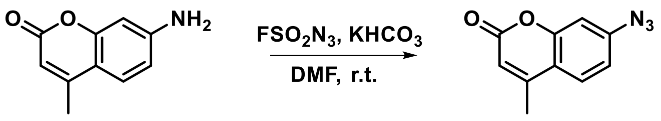

7

The synthesis of 7-azido-4-methylcoumarin was executed in accordance with a standard procedure, yielding an impressive 88%.

^{1}H NMR (**7**, 300 MHz, $CDCl_3$): δ_H 2.35 (3H, *d*, H-e), 5.00 (1H, *s*, H-c), 6.02 (1H, *s*, H-d), 6.55 (1H, *d*, H-a), 7.35 (1H, *d*, H-b). ESI-MS (*m/z*): calculated for $C_{10}H_7N_3O_2$ $[M+Na]^+$ 224.2, found 224.2.

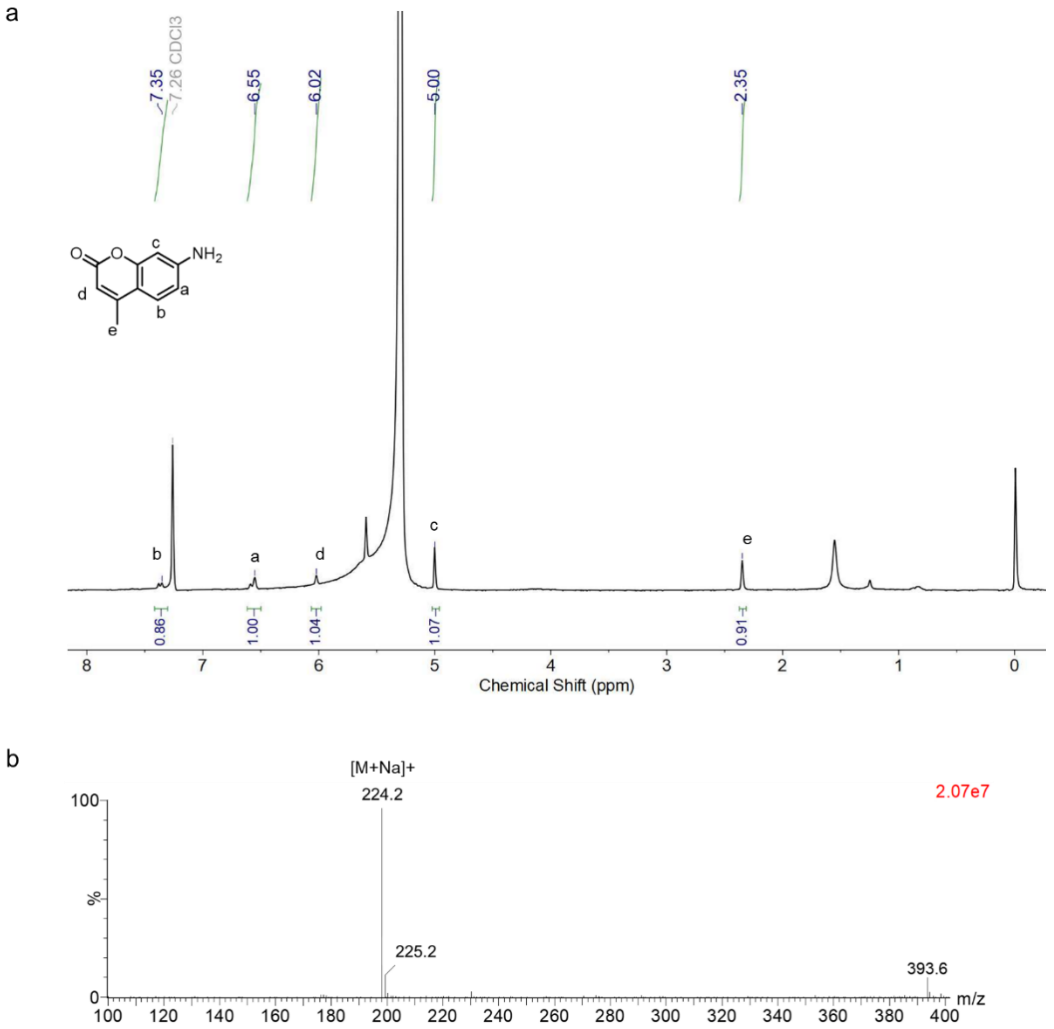

Figure S3. (a) ^{1}H NMR spectrum of 7-azido-4-methylcoumarin in $CDCl_3$. (b) ESI-MS (ESI^+) spectrum of 7-azido-4-methylcoumarin.

Synthesis of Compound 9

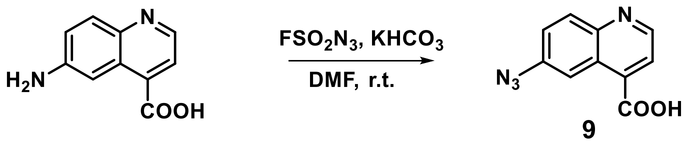

9

The synthesis of 6-azidoquinoline-4-carboxylic acid was executed in accordance with a standard procedure, yielding an impressive 80%.

^{1}H NMR (**9**, 300 MHz, DMSO-d$_6$): δ_H 7.60 (*q*, 1H, H-c), 8.00 (*d*, 1H, H-a), 8.17 (*d*, 1H, H-d), 8.50 (1H, *d*, H-b), 9.00 (1H, *d*, H-e), 13.90 (1H, *s*, -COOH). ESI-MS (*m/z*): calculated for $C_{10}H_6N_4O_2$ $[M\text{-}H]^-$ 213.1, found 213.1.

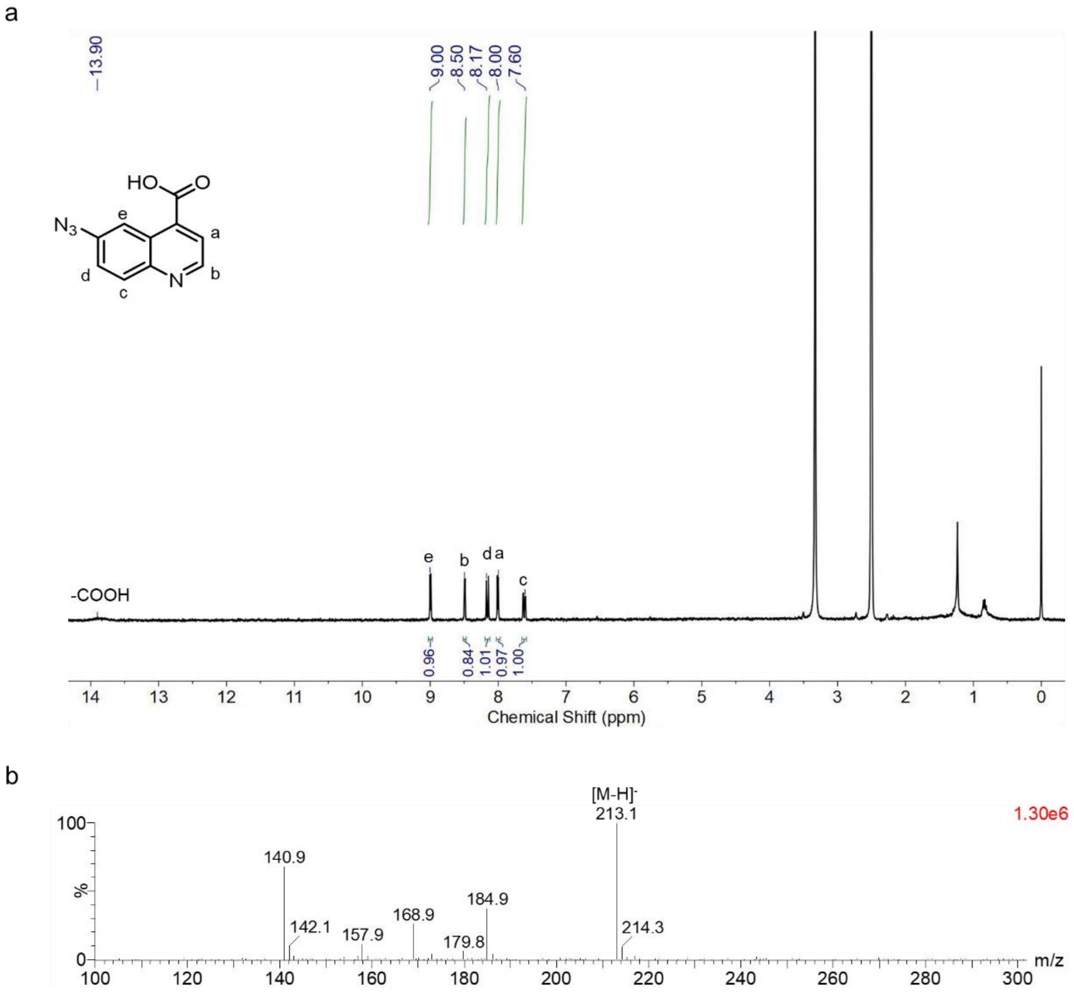

Figure S4. (a) ^{1}H NMR spectrum of 6-azidoquinoline-4-carboxylic acid in DMSO-d$_6$. (b) ESI-MS (ESI$^-$) spectrum of 6-azidoquinoline-4-carboxylic acid.

Synthesis of Compound 11

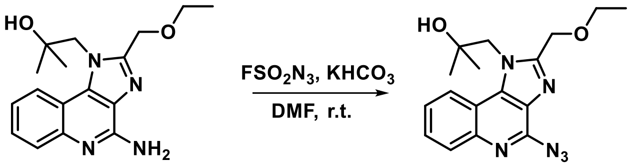

11

The synthesis of R848-N_3 was executed in accordance with a standard procedure, yielding an impressive 86%.

^{1}H NMR (**11**, 300 MHz, $CDCl_3$): δ_H 1.24 (3H, *t*, H-a), 1.40 (6H, *s*, H-e), 3.65 (2H, *q*, H-b), 4.87 (2H, *s*, H-d), 5.04 (2H, *s*, H-c), 7.76 (2H, *m*, H-f, H-i), 8.42 (1H, *d*, H-h), 8.83 (1H, *d*, H-g). ESI-MS (*m/z*): calculated for $C_{17}H_{20}N_6O_2$ $[M+H]^+$ 341.2, found

a

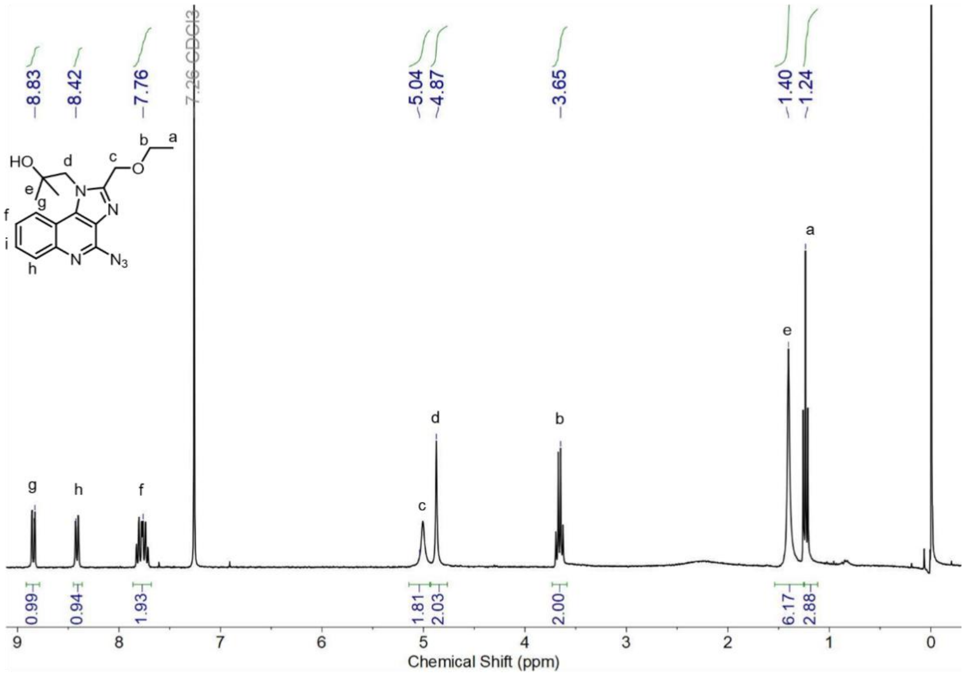

b

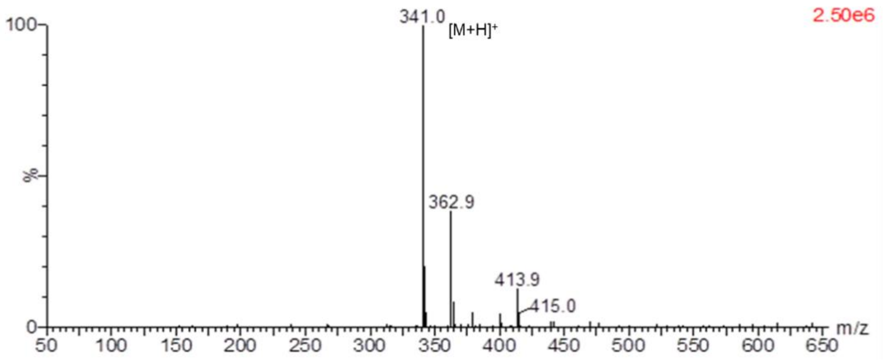

341.0.

Figure S5. (a) ^{1}H NMR spectrum of R848-N_3 in $CDCl_3$. (b) ESI-MS (ESI^{-}) spectrum of R848-N_3.

Synthesis of Compound 13

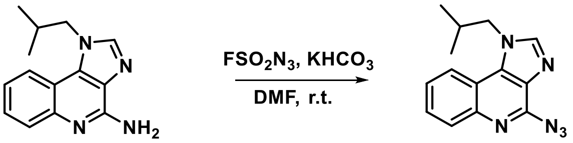

13

The synthesis of R837-N_3 was executed in accordance with a standard procedure, yielding an impressive 79%.

^{1}H NMR (**13**, 300 MHz, CF_3COOD): δ_H 1.07 (6H, *d*, H-a), 2.35 (1H, *m*, H-b), 4.41 (2H, *d*, H-c), 7.79 (2H, *m*, H-f, H-g), 8.00 (1H, *s*, H-d), 8.15 (1H, *m*, H-h), 8.86 (1H, *d*,

a

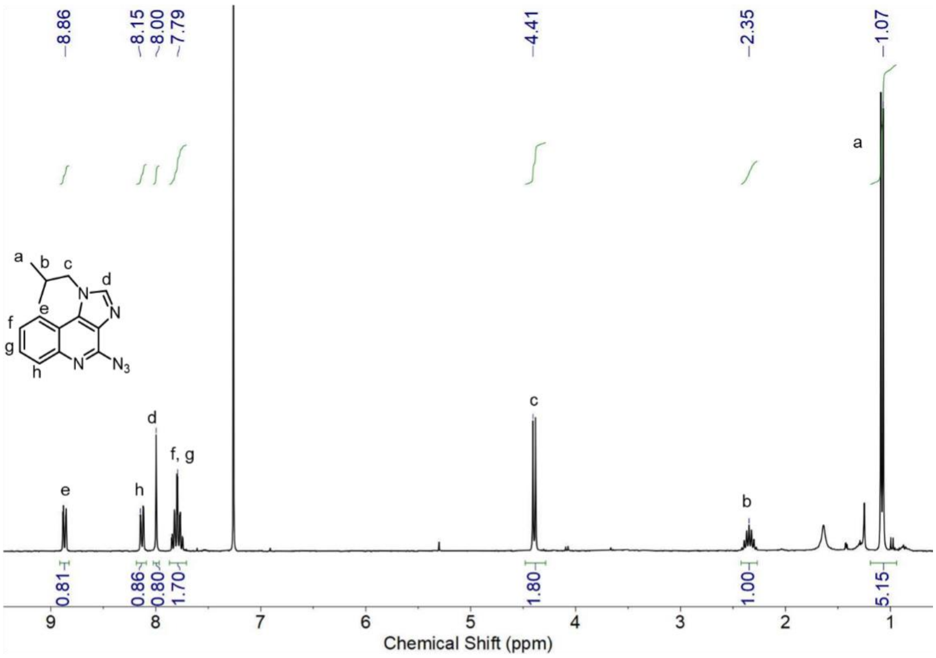

b

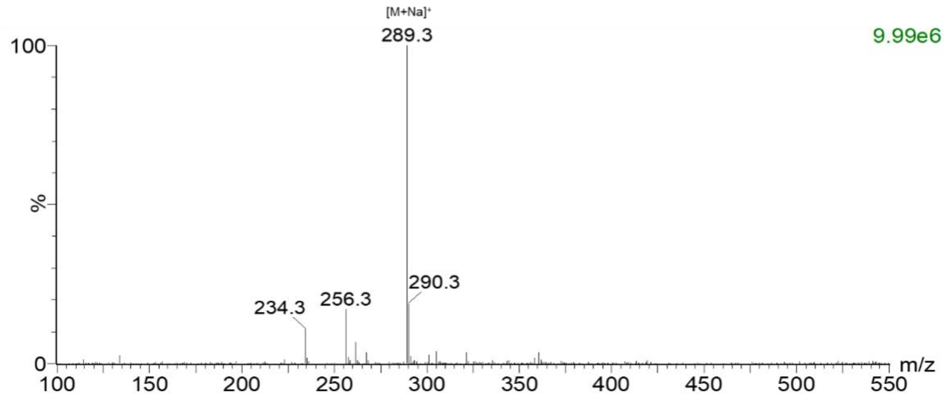

H-e). ESI-MS (*m/z*): calculated for $C_{14}H_{14}N_6$ $[M+Na]^+$ 289.2, found 289.3.

Figure S6. (a) ^{1}H NMR spectrum of R837-N_3 in CF_3COOD. (b) ESI-MS (ESI^{-}) spectrum of R837-N_3.

Synthesis of Compound 14

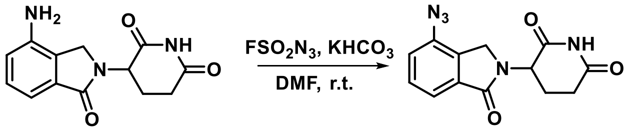

14

The synthesis of lenalidomide-N_3 was executed in accordance with a standard procedure, yielding an impressive 83%.

^{1}H NMR (**14**, 300 MHz, DMSO-d$_6$): δ_H 2.01, 2.39 (2H, *m*, H-b), 2.57, 2.87 (2H, *m*, H-c), 5.12 (1H, *t*, H-d), 5.75 (2H, *s*, H-e), 7.59 (3H, *m*, H-f, H-g, H-h), 10.99 (1H, *s*, H-a). ESI-MS (*m/z*): calculated for $C_{13}H_{11}N_5O_3$ [M+Na]$^+$ 308.2, found 308.2.

a

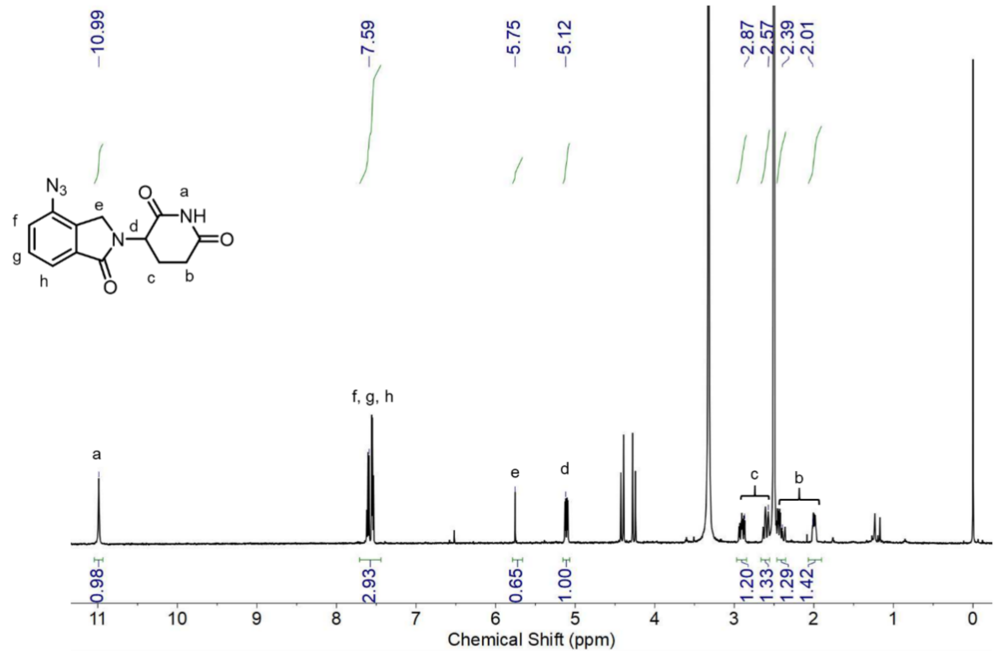

b

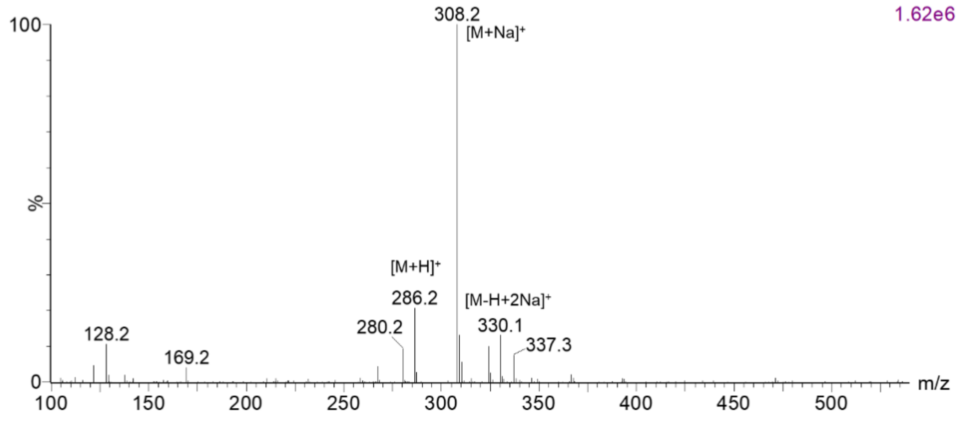

Figure S7. (a) ^{1}H NMR spectrum of lenalidomide-N_3 in DMSO-d_6. (b) ESI-MS (ESI^-) spectrum of lenalidomide-N_3.

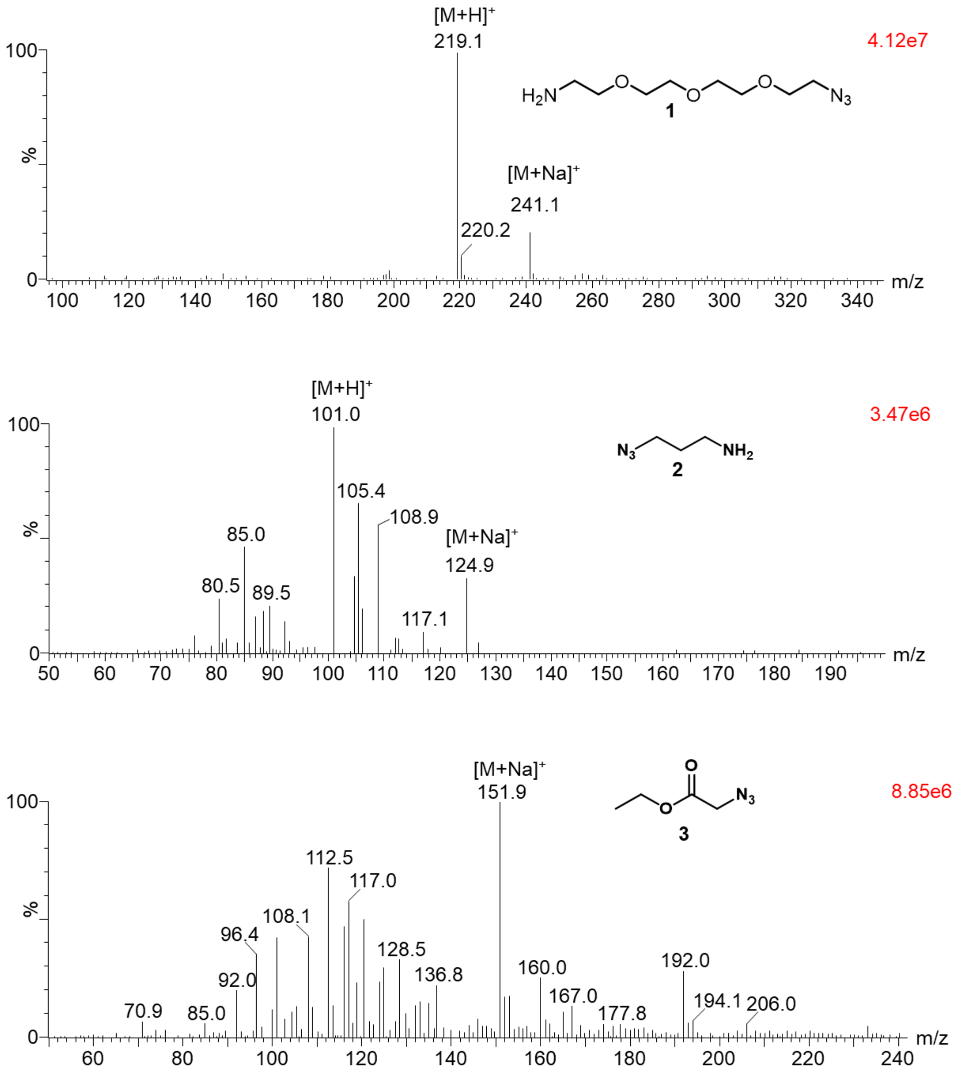

Figure S8. ESI-MS (ESI$^+$) spectrum of the aliphatic azide-containing compounds **1** (calculated for $C_8H_{18}N_4O_3$ $[M+H]^+$ 219.1, found 219.1), **2** (calculated for $C_3H_8N_4$ $[M+H]^+$ 101.1, found 101.0), and **3** (calculated for $C_4H_7N_3O_2$ $[M+Na]^+$ 152.0, found 151.9) after US stimulation (US: 1.0 MHz, 50% duty cycle and 2.5 W/cm^2, 5 min).

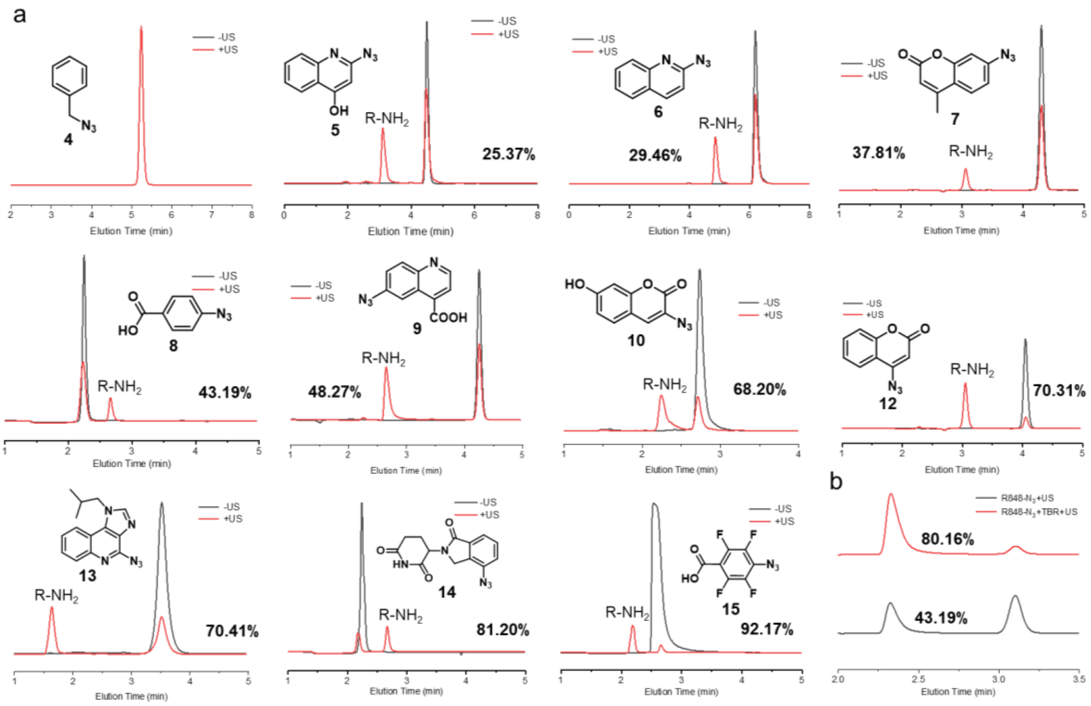

Figure S9. (a) HPLC curves of azide-containing compounds **4-15** after US stimulation. (US:1.0 MHz, 50% duty cycle and 2.5 W/cm^2, 5 min). (b) HPLC curves of R848-N_3 (1 mM, 1 equiv.) and riboflavin tetrabutyrate (TBR) (2 mM, 2 equiv.) under US conditions for 5 min in pH=5.5 buffer.

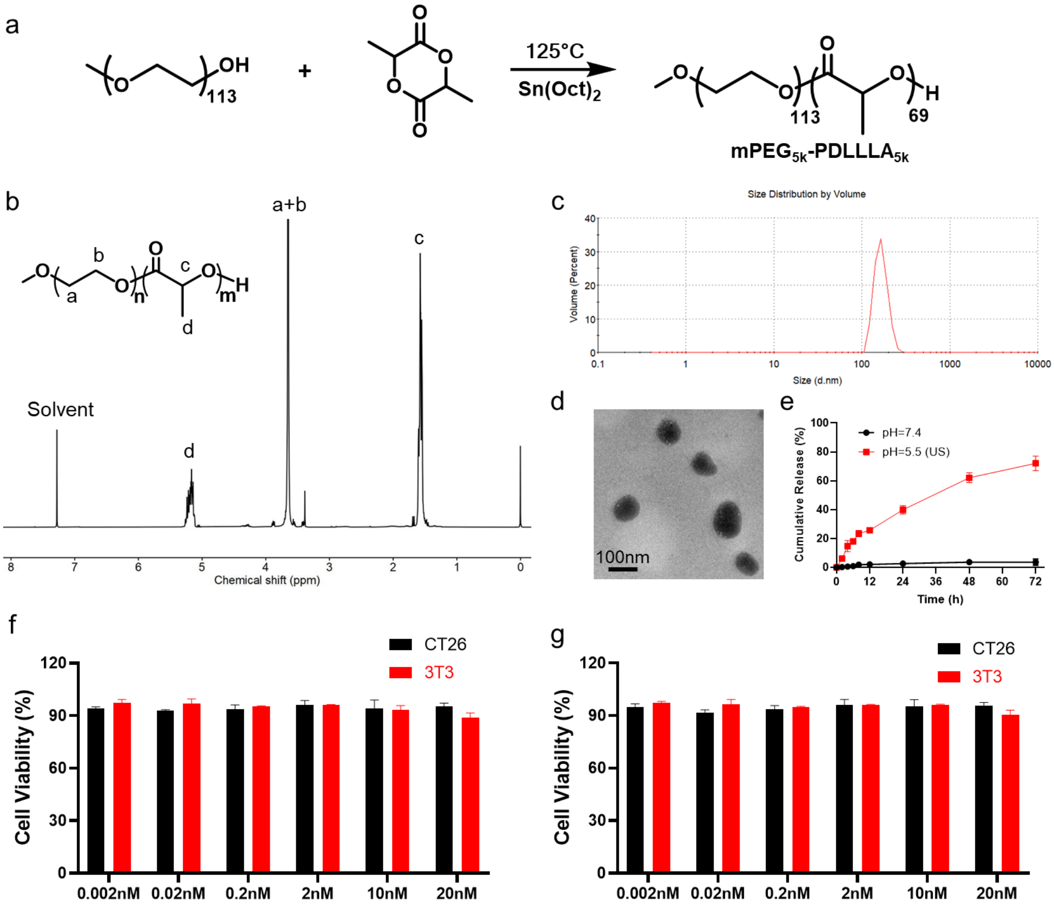

Figure S10. (a) Synthesis route of mPEG-PDLLA. (b) ^{1}H NMR of mPEG$_{5k}$-PDLLA$_{5k}$ in $CDCl_3$. (c) The hydrodynamic diameter distribution of NPs(R848-N_3+TBR) in aqueous solution as determined by DLS. (d) Transmission electron microscopy (TEM) images of NPs(R848-N_3+TBR). (e) Cumulative R848-N_3 release from NPs(R848-N_3+TBR) in pH = 7.4 or pH = 5.5 + US (1.0 MHz, 50% duty cycle and 2.5 W/cm^2, 2 min). In vitro cytotoxicities of CT26 and 3T3 cells after incubated with different concentrations of R848 (f) and R848-N_3 (g) for 24 h (n = 3).

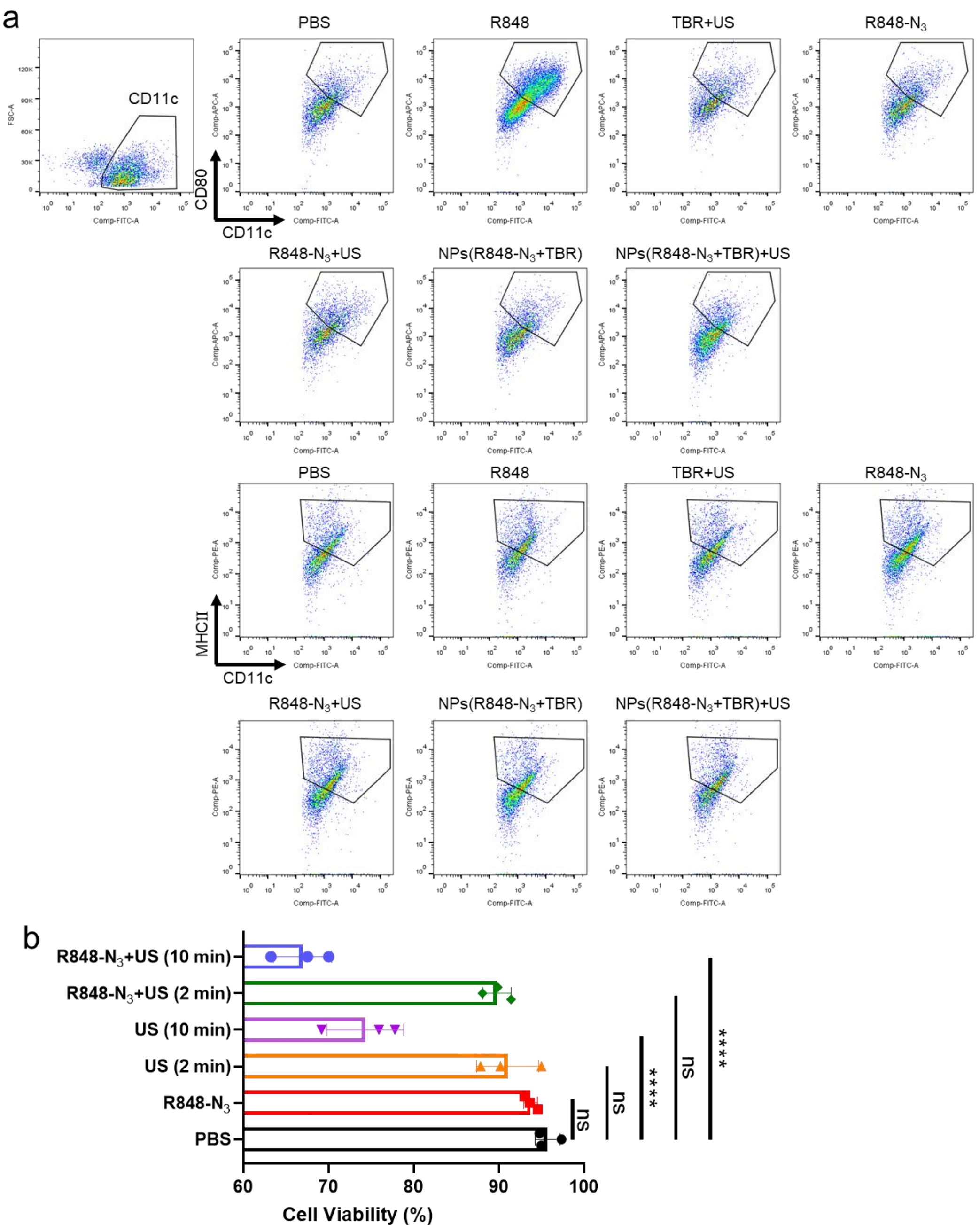

Figure S11. (a) The analysis of BMDCs activation was conducted using flow cytometry, accompanied by representative images of the same. The gating strategies employed for activated DCs included the identification of $CD11c^+CD80^+$ and $CD11c^+MHCII^+$ populations. (b) In vitro cytotoxicities of DCs treated with US (1.0 MHz, 50% duty cycle, 1.5 W/cm^2, 2 min or 10 min) after incubated with R848-N_3 for 24 h (n = 3). Statistical analysis was performed using one-way ANOVA with the corresponding Tukey's multiple comparison test. ****$P < 0.0001$; ns, not significant.

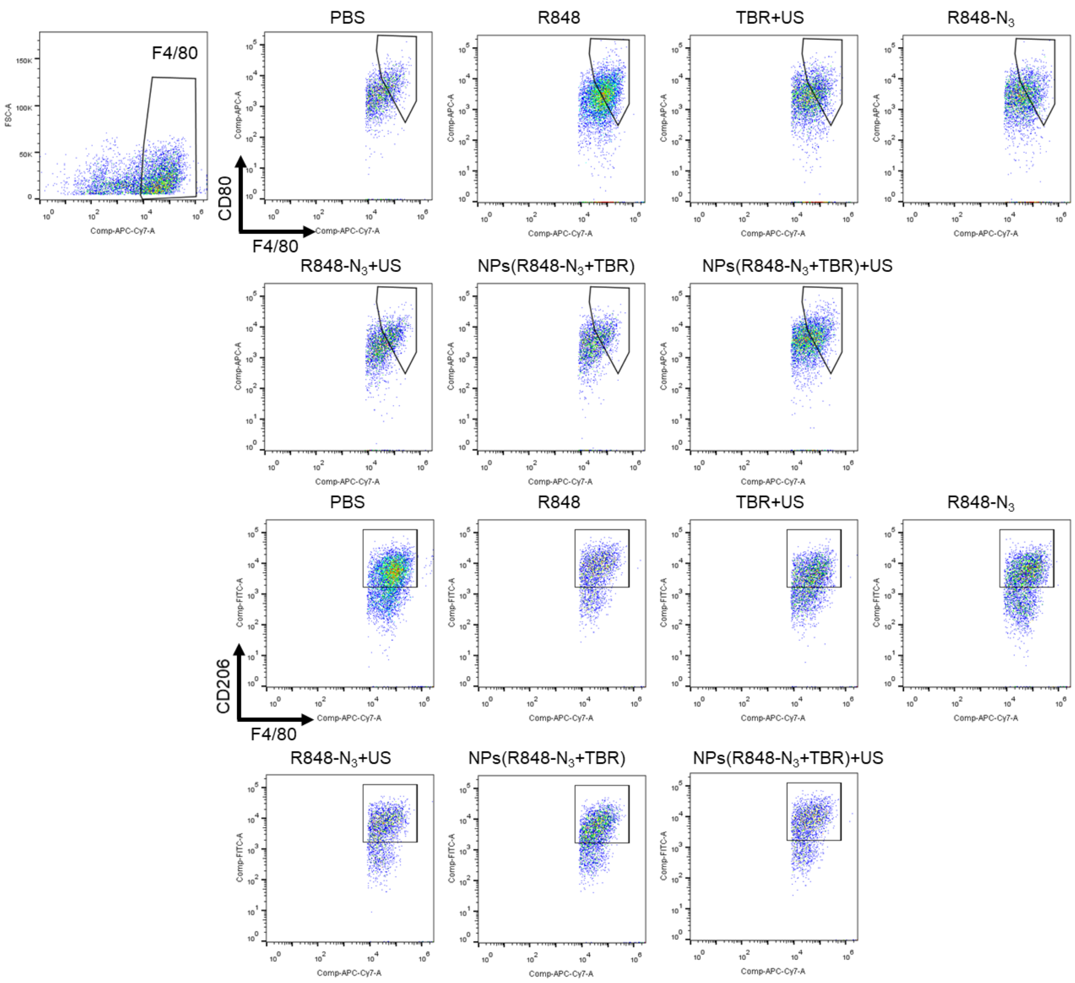

Figure S12. The analysis of BMDMs polarization was conducted using flow cytometry, accompanied by representative images of the same. The gating strategies employed for M1-like macrophages (F4/80$^+$CD80$^+$ population) and M2-like macrophages (F4/80$^+$CD206$^+$ population).

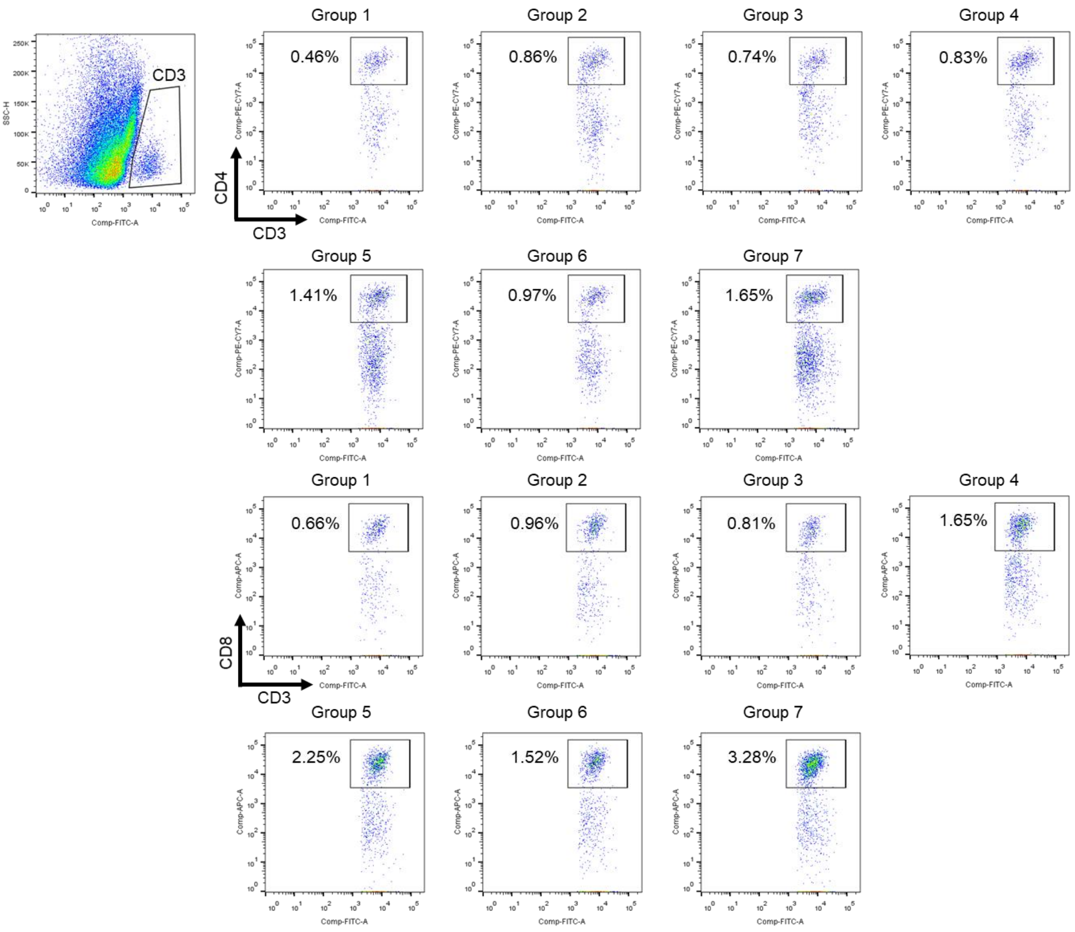

Figure S13. Flow cytometry results showing the percentage and gating strategies for $CD4^+$ T cells and $CD8^+$ T cells in the murine CT26 model following various treatments (*n*=5). The groups are delineated as follows: PBS (Group 1), R848-N_3 (Group 2), TBR+US (Group 3), R848-N_3+US (Group 4), R848-N_3+TBR+US (Group 5), NPs(R848-N_3+TBR) (Group 6), and NPs(R848-N_3+TBR) +US (Group 7).

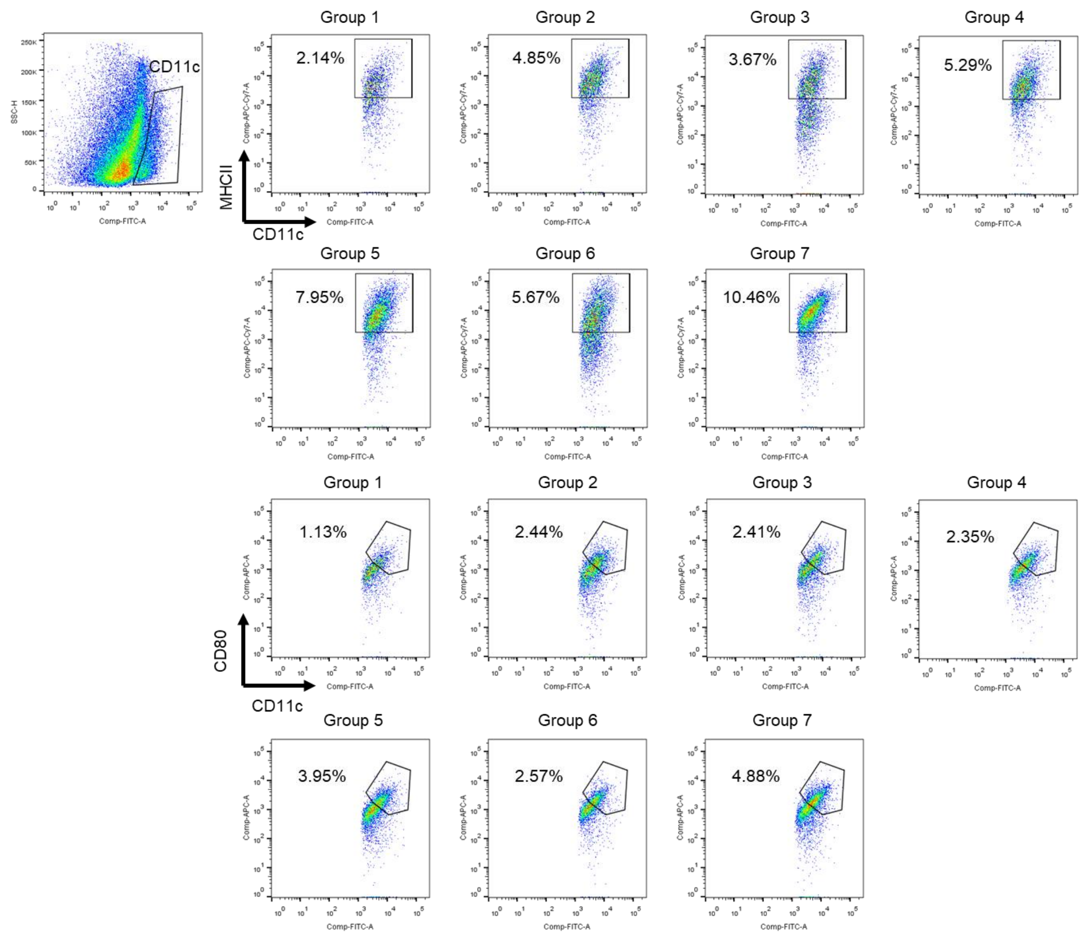

Figure S14. Flow cytometry results showing the percentage and gating strategies for activated DCs ($CD11c^{+}MHCII^{+}$ or $CD11c^{+}CD80^{+}$) in the murine CT26 model following various treatments (*n*=5). The groups are delineated as follows: PBS (Group 1), R848-N_3 (Group 2), TBR+US (Group 3), R848-N_3+US (Group 4), R848-N_3+TBR+US (Group 5), NPs(R848-N_3+TBR) (Group 6), and NPs(R848-N_3+TBR) +US (Group 7).

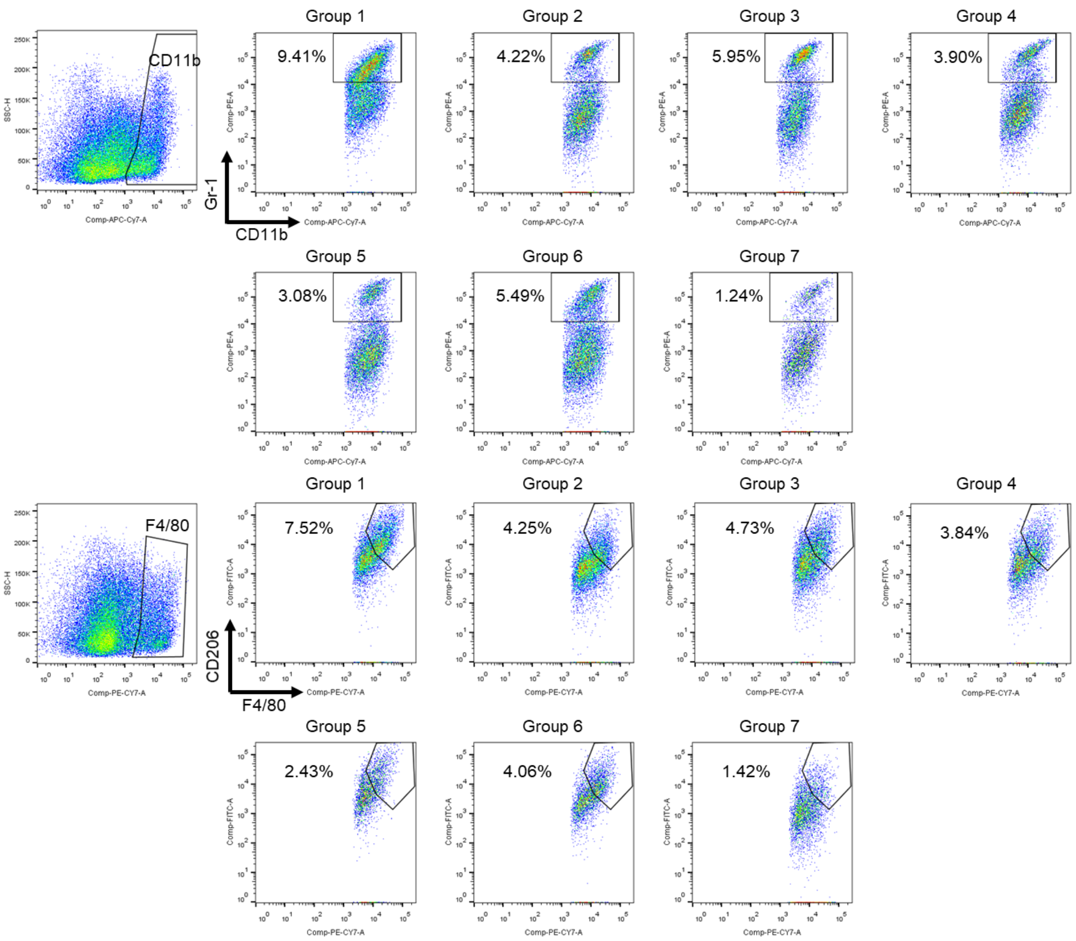

Figure S15. Flow cytometry results showing the percentage and gating strategies for activated MDSCs ($CD11b^+Gr\text{-}1^+$) and M2-like macrophages ($F4/80^+CD206^+$) in the murine CT26 model following various treatments (n=5). The groups are delineated as follows: PBS (Group 1), R848-N_3 (Group 2), TBR+US (Group 3), R848-N_3+US (Group 4), R848-N_3+TBR+US (Group 5), NPs(R848-N_3+TBR) (Group 6), and NPs(R848-N_3+TBR) +US (Group 7).

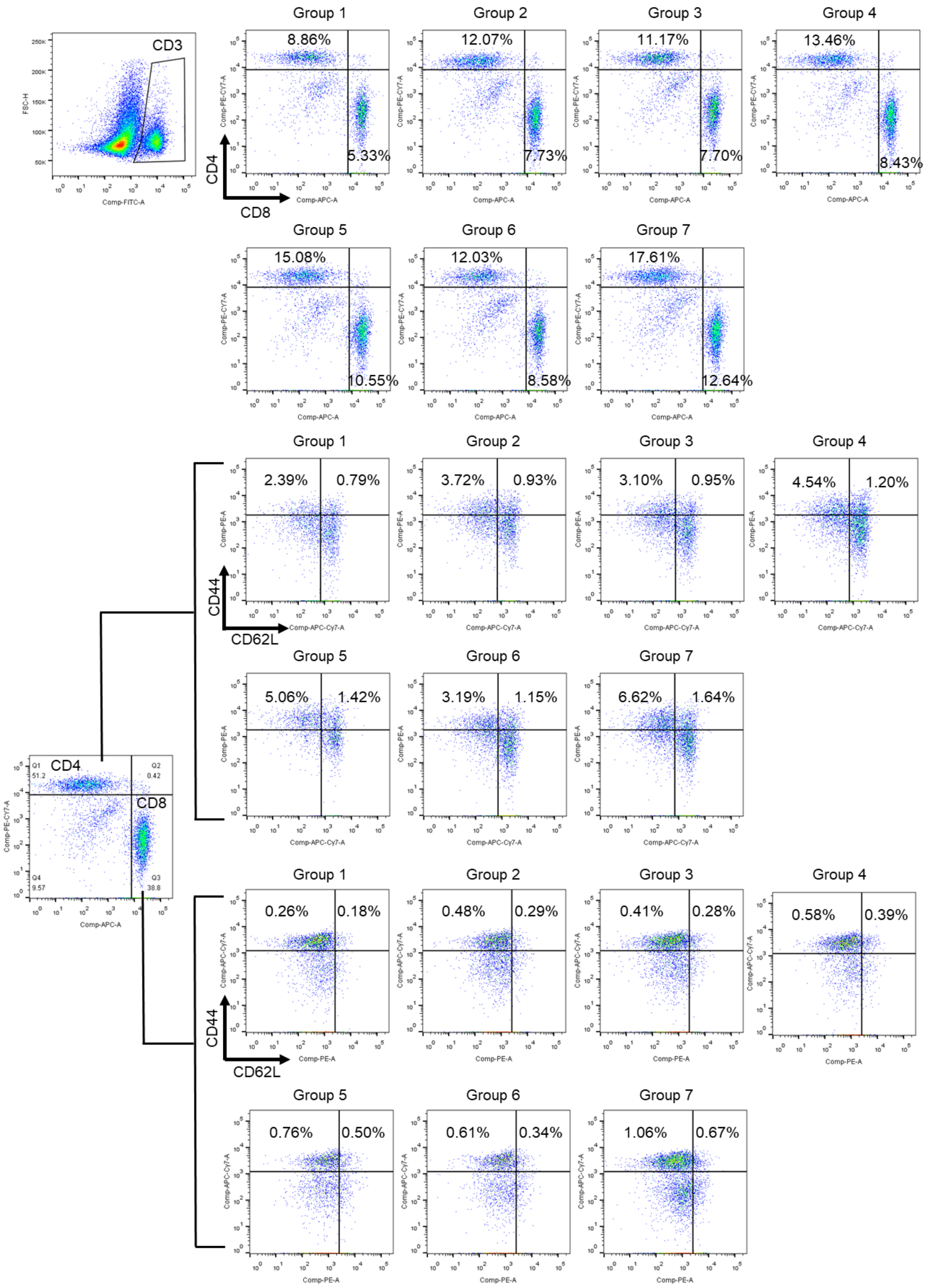

Figure S16. Flow cytometry results showing the percentage and gating strategies for CD4^{+} T cells, CD8^{+} T cells, CD4^{+} effector memory T cells (CD44^{+}CD62L^{-}), CD4^{+} central memory T cells (CD44^{+}CD62L^{+}), CD8^{+} effector memory T cells (CD44^{+}CD62L^{-}) and CD8^{+} central memory T cells (CD44^{+}CD62L^{-}) in the spleen following various treatments (*n*=5). The groups are delineated as follows: PBS

(Group 1), R848-N_3 (Group 2), TBR+US (Group 3), R848-N_3+US (Group 4), R848-N_3+TBR+US (Group 5), NPs(R848-N_3+TBR) (Group 6), and

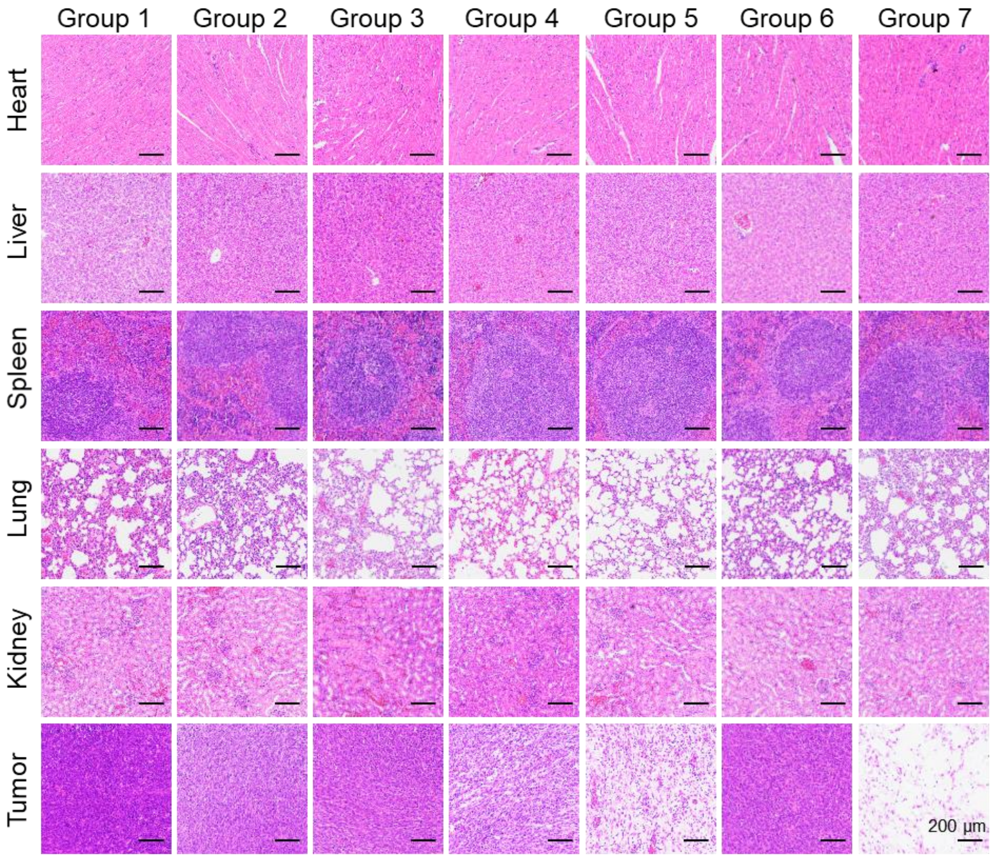

NPs(R848-N_3+TBR) +US (Group 7).

Figure S17. Hematoxylin and eosin staining of different organs (heart, liver, spleen, lung, and kidney) and tumors after various treatments (Scale bar: 100 μm). The groups are delineated as follows: PBS (Group 1), R848-N_3 (Group 2), TBR+US (Group 3), R848-N_3+US (Group 4), R848-N_3+TBR+US (Group 5), NPs(R848-N_3+TBR) (Group 6), and NPs(R848-N_3+TBR)+US (Group 7).

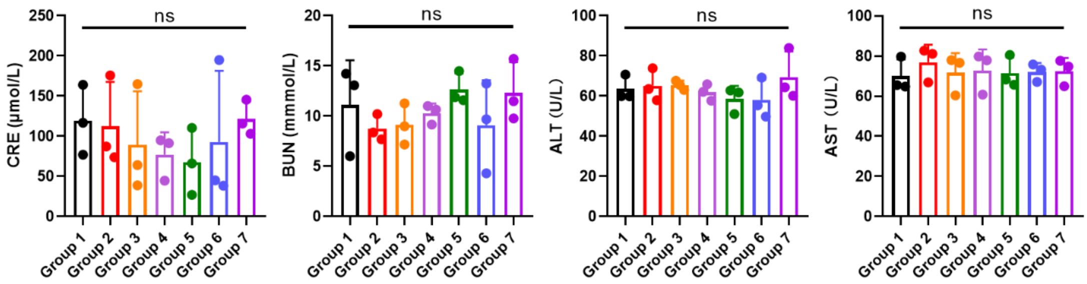

Figure S18. Biochemical analysis of blood was conducted on CT26 tumor-bearing mice subjected to various treatments. The groups are delineated as follows: PBS (Group 1), R848-N_3 (Group 2), TBR+US (Group 3), R848-N_3+US (Group 4), R848-N_3+TBR+US (Group 5), NPs(R848-N_3+TBR) (Group 6), and NPs(R848-N_3+TBR) +US (Group 7). Statistical analysis was performed using one-way ANOVA with the corresponding Tukey's multiple comparison test. ns, not significant.

Table S1. The values of reactions energy (ΔE), reaction enthalpy (ΔH) and Gibbs free energy (ΔG) are calculated by density functional theory (DFT) for the predicted reactions (DFT: B3LYP-D3(BJ)/def2-TZVP) at 1atm and 298.15K.

Reactions	ΔE (kcal/mol)	ΔH (kcal/mol)	ΔG (kcal/mol)
1.R848-N_3+NADH = R848-NH +NAD +N_2	-9.13	-13.10	-24.49
2.R848-NH +NADH = R848-NH_2+NAD ·	-26.37	-26.39	-26.58

Table S2. The ratios of R848 concentration in the tumor to other organs after treatment with R848-N_3.

Time	Tumor/Heart	Tumor/Liver	Tumor/Spleen	Tumor/Lung	Tumor/Kidney
1 h	6.28	1.86	4.31	4.15	1.99
4 h	3.78	1.05	2.38	4.00	1.56
10 h	2.01	0.87	1.57	1.82	1.62

Table S3. The ratios of R848 concentration in the tumor to other organs after treatment with R848-N_3+TBR+US.

Time	Tumor/Heart	Tumor/Liver	Tumor/Spleen	Tumor/Lung	Tumor/Kidney

1 h	14.67	3.53	9.36	8.11	4.72
4 h	10.47	2.29	5.96	5.58	3.27
10 h	2.30	0.95	1.71	1.62	5.26

Table S4. The ratios of R848 concentration in the tumor to other organs after treatment with NPs(R848-N_3+TBR).

Time	Tumor/Heart	Tumor/Liver	Tumor/Spleen	Tumor/Lung	Tumor/Kidney
1 h	7.66	1.77	5.63	4.92	1.92
4 h	8.76	4.18	3.57	7.63	1.44
10 h	9.96	7.19	4.65	9.94	3.22

Table S5. The ratios of R848 concentration in the tumor to other organs after treatment with NPs(R848-N_3+TBR)+US.

Time	Tumor/Heart	Tumor/Liver	Tumor/Spleen	Tumor/Lung	Tumor/Kidney
1 h	19.89	4.83	19.19	14.82	5.53
4 h	34.24	5.99	17.16	17.07	6.45
10 h	72.44	13.37	43.36	13.58	6.99

References

1. Meng G, Guo T, Ma T *et al*. Modular click chemistry libraries for functional screens using a diazotizing reagent. *Nature*. 2019; **574**: 86-9.

2. Sun J, Wei Q, Shen N *et al*. Predicting the loading capability of mPEG-PDLLA to hydrophobic drugs using solubility parameters. *Chinese J. Chem*. 2020; **38**: 690-6.
